# Supplementary material for: Scalable solution for delivery of diabetes self-management education in Thailand (DSME-T): a cluster randomised trial study protocol
Source: BMJ Open. 2020 Oct 5;10(10):e036963. doi: 10.1136/bmjopen-2020-036963 (PMC7537447; doi:10.1136/bmjopen-2020-036963)
Supplement: Supplementary data [file bmjopen-2020-036963supp001.pdf]

Participant Information Sheet

Title of Project: Delivering Diabetes Self-Management Education in Thailand

Introduction
We would like to invite you to take part in a research study. Joining the study is entirely up to you. Before you decide, you need to understand why the research is being done and what it would involve. One of our team will go through this information sheet with you, and answer any questions you may have. Ask questions if anything you read is not clear or you would like more information. Please feel free to talk to others about the study if you wish. Take time to decide whether or not to take part.

What is the purpose of the study?
The London School of Hygiene and Tropical Medicine (LSHTM) are conducting research for people with type 2 diabetes in Chiang Mai Thailand who have recently been diagnosed. The purpose of this study is to provide diabetes self-management education (DSME) by trained nurses and peer health care volunteers for those recently diagnosed.

This is a 3 arm cluster randomised trial where your service provider has been randomised to one of the following 3 interventions: 1) usual treatment 2) Nurse led DSME 3) Peer led DSME.

Why have I been asked to take part?
You have been invited because your healthcare provider is involved in the study and has identified you as being newly diagnosed with type 2 diabetes.

Do I have to take part?
No. It is up to you to decide to take part or now. If you don't want to take part, that's ok. Your doctor will still care for you and your decision will not affect the quality of care you receive.

We will discuss the study together and give you a copy of this information sheet. If you agree to take part, we will then ask you to sign a consent form.

What will happen to me if I take part?

Enrolling you in the study

If you are interested in taking part the research assistant will go through the information sheet with you. If you are happy with the information provided you may complete the consent form.

At this point you will also have the opportunity to raise with the researcher any questions you might have about the study. You do not have to enter the study unless you feel completely happy with what you are being asked to do.

Angkurawaranon C, *et al. BMJ Open* 2020; 10:e036963. doi: 10.1136/bmjopen-2020-036963

Participating in a diabetes education programme

You will be invited to attend a diabetes self-management educational programme. This programme will consist of a number of sessions delivered as monthly meetings led by community health volunteers or nurses. Short films about living with diabetes will be shown to introduce key topics.

The education and skills training will be followed by an open discussion session to discuss common challenges and solutions, set lifestyle goals and seek advice.

Collecting information

If you are happy to proceed with the study the researcher will then ask a series of questions relating to your health. This will be through completing a questionnaire booklet. These baseline questions will take about twenty minutes to complete. We will also take blood samples as the beginning of the study from trained phlebotomists.

Each participant will be compensated 200 baht for each of the baseline, 6 month and 12 month visit.

Follow up

Six months and twelve months after you have entered the study we will contact you again to ask you a further set of questions and blood samples to see how you are feeling now.

How is taking part in the study different from usual care?

Additional support and education may be provided through the study through the self-management education programme.

Whilst you are taking part in the study you will continue to be looked after by your healthcare providers, as normal. No treatment will be withheld from you during the course of this study.

The researcher will want you to complete some questionnaires at the beginning of the study and after six and twelve months. You may be asked to take part in an interview with a researcher.

What are the possible risks and disadvantages?

It will take some time to complete the questionnaire, around twenty minutes to complete. If you agree to be further interviewed, then this will also mean time will be needed to attend the interview. You may find minor discomfort from the taking blood samples. We will have trained phlebotomists to take the blood sample.

What are the possible benefits?
We cannot promise the study will help you but the information we get from the study will help our knowledge and understanding of this research area within diabetes self-management education in Thailand.

Angkurawaranon C, *et al. BMJ Open* 2020; 10:e036963. doi: 10.1136/bmjopen-2020-036963

What if something goes wrong?
If you have a concern about any aspect of this study, you should ask to speak to the researchers who will do their best to answer your questions <+66(0)616852307>. If you remain unhappy and wish to complain formally, you can do this by contacting <if LSHTM is the sponsor: Patricia Henley at rgio@lshtm.ac.uk or +44 (0) 20 7927 2626>

The London School of Hygiene and Tropical Medicine holds insurance policies which apply to this study. If you experience harm or injury as a result of taking part in this study, you may be eligible to claim compensation.

Can I change my mind about taking part?
Yes. You can withdraw from the study at any time. You just need to tell your doctor that you don't want to be in the study anymore. Your doctor will still care for you.

If you withdraw from the study we will destroy all your identifiable samples/ tape recorded interviews, but we will need to use the data collected on you up to your withdrawal

Or

You can withdraw from treatment but keep in contact with us to let us know your progress. Information collected may still be used. Any stored blood or tissue samples that can still be identified as yours will be destroyed if you wish, or will continue to be stored for further research.

What will happen to information collected about me?
All information collected about you will be kept private. Only the study staff and authorities who check that the study is being carried out properly will be allowed to look at information about you. Data may be sent to other study staff in London or in Chiang Mai but this will be anonymised. This means that any information about you which leaves the hospital, will have your name and address removed so that you cannot be recognised.

Your doctor will send some details about you to the study team in Chiang Mai University who will store it securely. Your personal details will be kept in a different safe place to the other study information and will be destroyed within 10 years of the end of the study.

At the end of the project, the study data will be archived at Chiang Mai University. The data will be made available to other researchers worldwide for research and to improve medical knowledge and patient care. Your personal information will not be included and there is no way that you can be identified.

What will happen to the results of this study?
The study results will be published in a medical journal so that other doctors can learn from them. Your personal information will not be included in the study report and there is no way that you can be identified from it.

Who is organising and funding this study?
London School of Hygiene & Tropical Medicine is the sponsor for the research and they have full responsibility for the project including the collection, storage and analysis of your data.
This study is funded by the Medical Research Council UK and The Thailand Research Fund

Who has checked this study?
All research involving human participants is looked at by an independent group of people, called a Research Ethics Committee, to protect your interests. This study has been reviewed and given favourable opinion by The London

Angkurawaranon C, *et al. BMJ Open* 2020; 10:e036963. doi: 10.1136/bmjopen-2020-036963

School of Hygiene and Tropical Medicine Research Ethics Committee (<ref: 16113>). The Research Ethics Committee, Faculty of Medicine, Chiang Mai University (No 326/2018) has also reviewed the study and have agreed that it is okay for us to ask people to take part.

Further information and contact details

Thank you for taking time to read this information leaflet. If you think you will take part in the study please read and sign the consent form.

If you would like any further information, please contact Dr. Chaisiri Angkurawaranon, MD, who can answer any questions you may have about the study.

Contact details: Chaisiri Angkurawaranon, Department of Family Medicine, Faculty of Medicine, Chiang Mai University. Tel +66(0)616852307, Email: chaisiri.a@cmu.ac.th

What happens if new information becomes available during the study?

Sometimes during a study, new information becomes available about the treatment being studied. If this happens, the research team will tell you and discuss whether you want to continue in the study. If you decide to stop taking part in the study your usual care will continue. If you decide to continue in the study you may be asked to sign an updated consent form. If we think you should withdraw from the study, we will explain the reasons and arrange for your care to continue.

What happens when the study stops?

Very occasionally a study is stopped early. If this happens, the reasons will be explained to you.

Tissue studies:
What will happen to the samples I give?
Blood samples will be used to measure HbA1c and lipids, coordinating where possible with the annual routine tests offered to patients to reduce duplication. Blood will be drawn by a trained phlebotomist when participants come for interview and sent to laboratory for analysis. Data will be linked to the participant information using a unique respondent ID, which will be assigned to all study participants.

Blood samples will be sent to a laboratory within the Chiang Mai area. The results of the blood tests will be provided to your health care professional who will discuss with you if there are any issues.

We may use some of the samples collected for future studies. These will be anonymised when stored, and all future research using these samples will be reviewed by an independent ethics committee.

Angkurawaranon C, *et al*. *BMJ Open* 2020; 10:e036963. doi: 10.1136/bmjopen-2020-036963

CONSENT FORM FOR PARTICIPANT AND REPRESENTATIVE

Title of Project: A scalable solution for delivery of Diabetes Self-Management Education in Thailand

Name of PI/Researcher responsible for project: Chaisiri Angkurawaranon

**Statement**	**Please initial or thumbprint* each box**
I confirm that I have read and understood the information sheet dated 08.10.2018 (version 1.0) for the above named study. I have had the opportunity to consider the information, ask questions and have these answered satisfactorily.	
I understand that my consent is voluntary and that I am free to withdraw this consent at any time without giving any reason and without my medical care or legal rights being affected.	
I understand that relevant sections of my/the participant's medical notes and data collected during the study may be looked at by authorised individuals from Chiang Mai University, where it is relevant to my taking part in this research. I give permission for these individuals to have access to these records.	
I understand that data about/from me/the participant may be shared via a public data repository or by sharing directly with other researchers, and that I will not be identifiable from this information	
I understand that the tissue sample collected from me will be used to support other research in the future, and may be shared anonymously with other researchers, for their ethically-approved projects	
I give permission for a copy of this consent form, which contains my personal information, to be made available to the Trial Coordinating Centre for monitoring purposes only.	
I agree to my health care provider being informed of my participation in the study.	
I agree to me taking part in the above named study.	

Printed name of participant/Representative	Signature of participant/Representative (or thumbprint/mark if unable to sign)	Date
Printed name of person obtaining consent	Signature of person obtaining consent	Date

The participant is unable to sign. As a witness, I confirm that all the information about the trial was given and the participant/representative consented to taking part *(*only required if the participant/representative is unable to read or write)*

Printed name of impartial witness*	Signature of impartial witness*	Date

A copy of this informed consent document has been provided to the participant.

Centre Number:
Study Number:
Participant Identification Number:

[Informed Consent for Participant and Representative for incapacitated adults_08_10_2018_V_1.0]

แบบขอรับความยินยอมเข้าร่วมโครงการ (Consent ข้อมูลสำหรับผู้ป่วย)

ชื่อโครงการศึกษาวิจัย : "การพัฒนาทางออกเพื่อการขยายการบริการที่เพิ่มความสามารถในการดูแลตนเองของผู้ที่เป็นเบาหวานในประเทศไทย"

หมายเลขโครงการศึกษาวิจัย : FAM-2561-05594

ผู้ให้ทุนสนับสนุนการวิจัย : สำนักงานคณะกรรมการส่งเสริมวิทยาศาสตร์ วิจัยและนวัตกรรม (สกสว.)

แพทย์ผู้วิจัยหลัก : อ.ดร.นพ.ชัยสิริ อังกุระวรานนท์

ท่านได้รับการเชื้อเชิญให้เข้าร่วมการศึกษาวิจัยนี้เนื่องจากท่านได้รับการวินิจฉัยว่าเป็นโรคเบาหวานที่เพิ่งได้รับการวินิจฉัยมาไม่เกิน 1 ปี หรือเป็นผู้ป่วยเบาหวานที่ยังไม่สามารถควบคุมน้ำตาลได้ ขอให้ท่านกรุณาอ่านข้อมูลข้างล่างก่อน (หรือผู้วิจัยได้อ่านให้ท่านรับทราบ) หากท่านมีข้อข้องใจใดๆ เกี่ยวกับการศึกษาวิจัยนี้ และสิทธิของท่าน กรุณาซักถามจากแพทย์ผู้ทำการศึกษาวิจัย หรือ ผู้ช่วยแพทย์ที่ทำการศึกษาวิจัย ซึ่งจะเป็นผู้สามารถให้ความกระจ่างแก่ท่านได้ หากท่านตัดสินใจเข้าร่วมการศึกษาวิจัย ท่านจะได้รับเอกสารข้อมูลสำหรับผู้เข้าร่วมโครงการวิจัยและสำเนาใบยินยอมที่ท่านเซ็นชื่อกำกับเก็บไว้ 1 ฉบับ

การศึกษาวิจัยนี้เกี่ยวกับเรื่องอะไร

เนื่องจากในปัจจุบันนี้มีผู้ป่วยเป็นโรคเบาหวานจำนวนมากขึ้น และพบว่าการป่วยเป็นโรคเบาหวานนี้สัมพันธ์กับภาวะแทรกซ้อนทางสุขภาพมากมาย เพื่อให้เป็นประโยชน์ต่อการพัฒนาการดูแลรักษาผู้ป่วยเบาหวานในอนาคต ผู้วิจัยจึงสนใจที่จะศึกษาว่าการเป็นโรคเบาหวานนั้นสัมพันธ์กับการเปลี่ยนแปลงระดับชีวภาพในร่างกายอย่างไร โดยผ่านการตรวจเลือดและอุจจาระ และเพื่อศึกษาว่าการจัดทำโครงการให้ความรู้ในการดูแลตนเองของผู้ป่วยเบาหวานโดยใช้สื่อมัลติมีเดียเข้าช่วยเหลือมีผลลัพธ์ของการรักษาที่ดีขึ้นได้หรือไม่ การศึกษานี้จะรวบรวมผู้ป่วยประมาณ 600 ราย จากโรงพยาบาลชุมชนที่ให้การรักษาแบบปฐมภูมิ ในจังหวัดเชียงใหม่ และ ลำปาง

ท่านจะต้องปฏิบัติตัวอย่างไร

หากท่านตัดสินใจเข้าร่วมการศึกษาวิจัยนี้ท่านจะถูกขอร้องให้เซ็นชื่อลงในใบยินยอมท่านจะได้รับการซักประวัติเกี่ยวกับโรคประจำตัวและการรักษาที่ได้รับ ได้ตรวจร่างกาย และการเจาะเลือด

เพื่อเป็นข้อมูลเริ่มต้นก่อนการรักษา โดยทั้งนี้ท่านจะได้รับการดูแลตามปกติควบคู่ไป

จากนั้นท่านจะได้รับการอบรมเพื่อพัฒนาศักยภาพในการดูแลตนเองในโรคเบาหวานของท่านโดยทีมดูแลสุขภาพ ซึ่งหน่วยบริการของท่านจะถูกสุ่มให้อยู่ 1 ใน 3 กลุ่ม อันได้แก่ กลุ่มทดลอง1 กลุ่มทดลอง2 และกลุ่มควบคุม โดยทั้ง 3 กลุ่มจะได้รับข้อมูลและการดูแลพื้นฐานเช่นเดียวกัน แต่แตกต่างกันในรูปแบบของการให้การอบรมในการดูแลตนเองของผู้ป่วยเบาหวาน ซึ่งจะใช้เวลานาน ไม่เกิน ครึ่งวัน โดย

Consent form WP3 intervention phase DSME 5 June 2018

- กลุ่มที่1 อบรมโดยบุคลากรทางการแพทย์ พยาบาล หรือ เจ้าหน้าที่สาธารณสุข
- กลุ่มที่2 อบรมโดยกลุ่มเพื่อนช่วยเพื่อนหรืออสม.ที่ผ่านการฝึกอบรมด้านการเพิ่มศักยภาพในการดูแลตนเองในโรคเบาหวาน
- กลุ่มที่ 3 ได้รับความรู้จากการดูแลตามมาตรฐานของกระทรวงสาธารณสุข

ซึ่งทั้งสามกลุ่ม จะมีการนัด หรือ ติดตาม ความก้าวหน้าพฤติกรรมการดูแลตนเอง ทุกๆ 1-3 เดือน

การติดตามผลเลือดและตอบแบบสอบถาม จะถูกทำในครั้งแรกข้างต้น และอีก 2 ครั้ง คือ ที่ 6 เดือนและที่ 12 เดือนนับจากครั้งแรกที่อบรม โดยทั้ง 3 ครั้ง ท่านจะถูกเจาะเลือดเพื่อส่งตรวจปริมาตรทั้งสิ้นครั้งละ 40 มิลลิลิตร

ความเสี่ยงจากการเข้าร่วมการวิจัยนี้

ความเสี่ยงจากการเจาะเลือด - ท่านอาจรู้สึกหน้ามืด เป็นลม ปวดบริเวณที่เจาะ หรือมีจ้ำเลือดบริเวณที่เจาะ มีความเสี่ยงน้อยมากที่จะเกิดการติดเชื้อจากการเจาะเลือดเพราะเราใช้เข็มเจาะเลือดที่ปราศจากเชื้อและใช้ครั้งเดียวทิ้ง การเจาะเลือดทั้งหมดอยู่ในสถานพยาบาล และหากมีปัญหาจะได้รับการดูแลทันที

ท่านจะได้ประโยชน์อะไรจากการศึกษานี้

ท่านจะได้รับความรู้เพื่อนำไปใช้ในการดูแลตนเองเพื่อควบคุมโรคเบาหวานของท่าน และผลสรุปที่ได้จากการศึกษานี้จะเป็นประโยชน์ต่อผู้ป่วยรายอื่นในอนาคต

ค่าใช้จ่ายในการเข้าร่วมวิจัย

ท่านจะไม่ต้องเสียค่าใช้จ่ายเพิ่มเติมเกี่ยวกับการวิจัยนอกเหนือจากค่ารักษาปกติที่ควรจะเป็นซึ่งท่านสามารถใช้สิทธิในการเบิกจ่ายได้ตามสิทธิการรักษาปกติของท่าน

ค่าตอบแทน

สำหรับการมาตรวจติดตามที่เพิ่มจากการตรวจรักษาตามปกติ จำนวน 5 ครั้ง ซึ่งจะมีการตรวจเลือด รวม 3 ครั้ง ท่านจะได้รับค่าเดินทางและค่าเสียเวลาครั้งละ 100 บาท (รวม 500 บาทต่อคน ตลอดระยะเวลาโครงการ 1 ปี)

หากท่านได้รับบาดเจ็บจากการเข้าร่วมการศึกษาวิจัย

หากท่านได้ปฏิบัติตามคำแนะนำของแพทย์ผู้วิจัยแล้ว กระบวนการต่าง ๆ ในการวิจัยทำให้ท่านได้รับบาดเจ็บ เมื่อผู้วิจัยได้รับแจ้งจากท่าน ท่านจะได้รับการส่งต่อเพื่อรักษาภาวะดังกล่าวทันที

ท่านจะทำอย่างไรหากท่านไม่ต้องการเข้าร่วมการศึกษาวิจัย หรือเปลี่ยนใจระหว่างร่วมศึกษาวิจัย

ท่านไม่จำเป็นต้องเข้าร่วมการศึกษาวิจัยนี้หากท่านไม่สมัครใจ หลังจากท่านตัดสินใจจะเข้าร่วมการศึกษาแล้ว ท่านสามารถจะถอนตัวได้ตลอดเวลา การตัดสินใจของท่านจะไม่มีผลต่อการรักษาในอนาคต หรือการดูแลอื่นใดหากท่านไม่ต้องการเข้าร่วมการศึกษาหรือต้องการหยุดการศึกษา ณ เวลาใดก็ตาม แพทย์ของท่านจะอธิบายให้ทราบถึงการรักษาอื่น ๆ ซึ่งเป็นทางเลือกที่มีอยู่ขณะนี้ ผู้วิจัยอาจจะตัดสินใจยกเลิกท่านจากการศึกษา หากเห็นว่าจะเป็นประโยชน์สำหรับท่านมากกว่า

ใครจะรู้บ้างว่าท่านเข้าร่วมการศึกษานี้

แพทย์ประจำตัวท่าน (แพทย์เวชปฏิบัติทั่วไป) ควรจะได้รับทราบว่าท่านตัดสินใจเข้าร่วมการศึกษาวิจัยนี้ ข้อมูลของท่านที่ถูกบันทึกไว้ระหว่างการศึกษา เช่นเดียวกับข้อมูลที่เกี่ยวข้องจากแฟ้มเวชระเบียนของโรงพยาบาล คลินิก บริษัทฯ หรือข้อมูลอื่น ๆ จะถูกเก็บไว้เป็นความลับตลอดเวลา คณะกรรมการจริยธรรมการวิจัยสามารถที่จะขอตรวจสอบข้อมูลเหล่านี้ได้ โดยข้อมูลเหล่านี้จะยังเก็บรักษาไว้เป็นเรื่องลับเฉพาะ

การปกป้องรักษาข้อมูล : ข้อมูลใดบ้างที่จะถูกเก็บรวบรวมไว้จากการศึกษานี้

ข้อมูลที่ถูกเก็บรวบรวมนั้นจะมีเฉพาะในส่วนที่เกี่ยวข้องกับการศึกษาเพื่อวัตถุประสงค์ทางการวิจัยทางการแพทย์ โดยจะไม่มีการอ้างถึงข้อมูลส่วนตัวของท่าน อันได้แก่ชื่อท่าน ในรายงานหรือวารสารใด ๆ หากท่านตกลงใจเข้าร่วมการศึกษา ท่านยินยอมที่จะไม่จำกัดการให้ข้อมูลที่เป็นส่วนตัวยกเว้นในกรณีที่ขัดต่อสิทธิส่วนบุคคลภายใต้กฎหมายคุ้มครองลิขสิทธิ์ส่วนบุคคล

หากท่านมีคำถามเกี่ยวกับการศึกษานี้ท่านสามารถติดต่อใครได้บ้าง

หากท่านมีคำถามหรือมีความวิตกกังวลเกี่ยวกับการศึกษาวิจัยนี้ หรือสงสัยว่าท่านกำลังได้รับบาดเจ็บจากการเข้าร่วมการวิจัยนี้ กรุณาติดต่อ คุณกุลญาภา อยู่นัด ได้ที่ ภาควิชาเวชศาสตร์ครอบครัว โทรศัพท์ที่ทำงาน 053-936362 (ในเวลาราชการ)

ส่วนแสดงความยินยอม

โดยการลงนามในหนังสือยินยอมฉบับนี้ ท่านยอมรับว่าได้อ่านเอกสารฉบับนี้แล้วและได้รับคำอธิบายเกี่ยวกับการศึกษาวิจัยนี้ รวมถึงได้รับคำตอบเกี่ยวกับข้อสงสัยต่าง ๆ ที่ท่านมีจากผู้วิจัยแล้ว

Consent form WP3 intervention phase DSME 5 June 2018

ท่านตกลงใจที่จะเข้าร่วมในการศึกษาวิจัยนี้ และมีสิทธิ์ที่จะถอนตัวจากการศึกษาวิจัยนี้ได้ทุกเมื่อ โดยไม่มีผลต่อการรักษาในอนาคต โดยการลงนามนี้ท่านไม่ได้สละสิทธิ์ใด ๆ ที่พึงมีทางกฎหมาย

ทั้งนี้ ท่านยินยอมที่จะเข้ารับการอบรมในโครงการวิจัย ตอบแบบสอบถามและ

ลายมือชื่ออาสาสมัคร ______________________________ วัน-เดือน-ปี_______________

(________________________________)

ลายมือชื่อเจ้าหน้าที่ผู้ให้ข้อมูล ______________________________ วัน-เดือน-ปี_______________
ณ สถานพยาบาล

(________________________________)

พยาน ______________________________ วัน-เดือน-ปี_______________

(________________________________)

Consent form WP3 intervention phase DSME 5 June 2018
