# Supplementary material for: Scalable solution for delivery of diabetes self-management education in Thailand (DSME-T): a cluster randomised trial study protocol
Source: BMJ Open. 2020 Oct 5;10(10):e036963. doi: 10.1136/bmjopen-2020-036963 (PMC7537447; doi:10.1136/bmjopen-2020-036963)
Supplement: Author's manuscript [file bmjopen-2020-036963.draft_revisions.pdf]

BMJ Open is committed to open peer review. As part of this commitment we make the peer review history of every article we publish publicly available.

When an article is published we post the peer reviewers' comments and the authors' responses online. We also post the versions of the paper that were used during peer review. These are the versions that the peer review comments apply to.

The versions of the paper that follow are the versions that were submitted during the peer review process. They are not the versions of record or the final published versions. They should not be cited or distributed as the published version of this manuscript.

BMJ Open is an open access journal and the full, final, typeset and author-corrected version of record of the manuscript is available on our site with no access controls, subscription charges or pay-per-view fees (http://bmjopen.bmj.com).

If you have any questions on BMJ Open's open peer review process please email
info.bmjopen@bmj.com

A Scalable Solution for Delivery of Diabetes Self-Management Education in Thailand: A Cluster Randomized Trial

Journal:	*BMJ Open*
Manuscript ID	bmjopen-2020-036963
Article Type:	Protocol
Date Submitted by the Author:	13-Jan-2020

Complete List of Authors:	Angkurawaranon, Chaisiri; Chiang Mai University, Department of Family Medicine, Faculty of Medicine<br>Papachristou Nadal, Iliatha; London School of Hygiene and Tropical Medicine, Faculty of Epidemiology and Population Health<br>Mallinson, Poppy; London School of Hygiene and Tropical Medicine Faculty of Epidemiology and Population Health, Department of Non-communicable Disease Epidemiology<br>Pinyopornpanish, Kanokporn; Chiang Mai University, Department of Family Medicine, Faculty of Medicine<br>Quansri, Orawan; Mahidol University<br>Rerkasem, Kittipan ; Chiang Mai University, Department of Surgery, Faculty of Medicine, Chiang Mai University; Chiang Mai University, Research Institute for Health Sciences<br>Srivanichakorn, Supattra; Royal Thai Government Ministry of Public Health<br>Techakehakij, Win; Lampang Hospital<br>Wichit, Nutchanath; Surat Thani Rajabhat University<br>Pateekhum, Chanapat; Chiang Mai University<br>Hashmi, Ahmar; Chiang Mai University, Department of Family Medicine<br>Hanson, Kara; London School of Hygiene and Tropical Medicine<br>Khunti, Kamlesh; University of Leicester, Department of Health Sciences<br>Kinra, Sanjay; London School of Hygiene and Tropical Medicine

Keywords:	DIABETES & ENDOCRINOLOGY, EDUCATION & TRAINING (see Medical Education & Training), Protocols & guidelines < HEALTH SERVICES ADMINISTRATION & MANAGEMENT

SCHOLARONE™
Manuscripts

I, the Submitting Author has the right to grant and does grant on behalf of all authors of the Work (as defined in the below author licence), an exclusive licence and/or a non-exclusive licence for contributions from authors who are: i) UK Crown employees; ii) where BMJ has agreed a CC-BY licence shall apply, and/or iii) in accordance with the terms applicable for US Federal Government officers or employees acting as part of their official duties; on a worldwide, perpetual, irrevocable, royalty-free basis to BMJ Publishing Group Ltd ("BMJ") its licensees and where the relevant Journal is co-owned by BMJ to the co-owners of the Journal, to publish the Work in this journal and any other BMJ products and to exploit all rights, as set out in our licence.

The Submitting Author accepts and understands that any supply made under these terms is made by BMJ to the Submitting Author unless you are acting as an employee on behalf of your employer or a postgraduate student of an affiliated institution which is paying any applicable article publishing charge ("APC") for Open Access articles. Where the Submitting Author wishes to make the Work available on an Open Access basis (and intends to pay the relevant APC), the terms of reuse of such Open Access shall be governed by a Creative Commons licence – details of these licences and which Creative Commons licence will apply to this Work are set out in our licence referred to above.

Other than as permitted in any relevant BMJ Author's Self Archiving Policies, I confirm this Work has not been accepted for publication elsewhere, is not being considered for publication elsewhere and does not duplicate material already published. I confirm all authors consent to publication of this Work and authorise the granting of this licence.

For peer review only

A Scalable Solution for Delivery of Diabetes Self-Management Education in Thailand: A Cluster Randomized Trial

Chaisiri Angkurawaranon[1*], Iliatha Papachristou Nadal[2,11], Poppy Mallinson[2,] Kanokporn Pinyopornpanish[1], Orawan Quansri[3], Kittipan Rerkasem[4,5], Supattra Srivanichakorn[6], Win Techakehakij[7], Nutchanath Wichit[8], Chanapat Pateekhum[1], Ahmar Hashmi[1], Kara Hanson[9], Kamlesh Khunti[10], Sanjay Kinra[2]

Author affiliations

[1] Department of Family Medicine, Faculty of Medicine, Chiang Mai University

[2] Faculty of Epidemiology and Population Health, London School of Hygiene and Tropical Medicine

[3] ASEAN Institute for Health Development, Mahidol University

[4] Department of Surgery, Faculty of Medicine, Chiang Mai University

[5] Research Institute for Health Sciences, Chiang Mai University

[6] Department of Disease Control, Ministry of Public Health

[7] Department of Social Medicine, Lampang Hospital

[8] Faculty of Nursing, Suratthanni Rajabhat University

[9] Faculty of Public Health and Policy, London School of Hygiene and Tropical Medicine

[10] Diabetes Research Center, University of Leicester

[11]Psychological Medicines, King's College London

Corresponding author: Chaisiri Angkurawaranon, Department of Family Medicine, Faculty of Medicine, Chiang Mai University, email: chaisiri.a@cmu.ac.th

For peer review only

For peer review only

For peer review only - http://bmjopen.bmj.com/site/about/guidelines.xhtml

ABSTRACT

Introduction Type 2 diabetes mellitus is amongst the foremost health challenges facing policy makers in Thailand as its prevalence has more than tripled over the last two decades, accounting for considerable death, disability and healthcare expenditure. Diabetes Self-Management Education (DSME) programmes shows promise in improving diabetes outcomes, but this is not routinely utilised in Thailand. This study aims to test a culturally tailored DMSE model in Thailand, using a 3-arm cluster randomised controlled trial comparing a nurse-led model, a peer-assisted model, and standard care. We will test which model is effective and cost effective to improve cardiovascular risk and control of blood glucose among people with diabetes.

Methods and analysis 21 primary care units in northern Thailand will be randomised to one of three interventions, enrolling a total of 693 patients. The primary care units will be randomised (1:1:1) to participate in a culturally-tailored DSME intervention for 12 months. The 3-arm trial design will compare effectiveness of nurse-led, peer-assisted (Thai village health volunteers) and standard care. The primary trial outcome is glycaemic control. A process evaluation and cost effectiveness evaluation will be conducted to produce policy relevant guidance for the Thai Ministry of Public Health. The planned trial period will start in January 2020 and finish October 2021.

Ethics and dissemination Ethical approval was submitted in Thailand and the UK. We will share our study data with other researchers, advertising via our publications and web presence. In particular, we are committed to sharing our findings and data with academic audiences in Thailand and other low- and middle-income countries.

For peer review only - http://bmjopen.bmj.com/site/about/guidelines.xhtml

Trial registration number:

ClinicalTrials.gov ID NCT03938233

Strengths and limitation of this study

- A three-arm cluster randomized control trial to evaluate clinical and cost-effectiveness of a culturally tailored DSME under two alternative modes of delivery (nurse-led and peer-assisted) will provide policy makers with options for scalability.
- A culturally-tailored DSME programme has been developed with input from stakeholders (policy makers, clinicians, nurses, village health volunteers and people with diabetes).
- A series of short films has been developed to introduce key topics, as there is increasing recognition that films are a highly efficient medium for communicating information, particularly in low literacy settings

For peer review only - http://bmjopen.bmj.com/site/about/guidelines.xhtml

INTRODUCTION

Type 2 diabetes mellitus (hereto referred to as diabetes) is amongst the foremost health challenges facing policy makers in Thailand. Its prevalence has more than tripled over the last two decades to an estimated 4 million adults (age adjusted prevalence 7.1%) living with diabetes in 2015.[1,2] Diabetes is associated with several macrovascular (e.g. ischaemic heart disease) and microvascular complications (e.g. nephropathy, retinopathy, neuropathy, and foot disease), which primarily account for the considerable death and disability (of which diabetes is the 5th leading cause in Thailand). In addition, diabetes in Thailand causes two-fold increase in healthcare expenditure and significant loss of economic productivity—of both diabetic patients and their carers.[1–4]

The complications of diabetes can be largely prevented or delayed through lifestyle change and medication when necessary, and regular screening for early detection and management of complications to control risk factors such as blood glucose, lipids and blood pressure.[3,4] Under Thailand's universal health coverage, nearly everyone diagnosed with diabetes receives timely medical care (>97%) and has access to screening. Yet, surveys suggest that only about half of the people with diabetes achieve optimal control of risk factors or receive annual screening for microvascular complications (53-60%).[1,5] Limited data support a lack of engagement and self-management skills among those diagnosed with diabetes as the main underlying reasons for this.[6]

Successful management of diabetes involves a considerable degree of self-management. People with diabetes need to adhere to multiple behaviours, including healthy lifestyles, regular monitoring and medication, problem-solving and healthy coping. In this, they are greatly supported by diabetes self-management education (DSME), defined as 'a collaborative and ongoing process intended to facilitate the development of knowledge, skills,

For peer review only - http://bmjopen.bmj.com/site/about/guidelines.xhtml

and abilities that are required for successful self-management of diabetes'.[7] Evidence from over 100 studies, including many randomised controlled trials, conducted predominantly in high-income countries, suggests that DSME programs are associated with improvements in a range of behavioural (knowledge, behaviours, self-efficacy, psychosocial) and clinical (physiological risk factors, screening for complications, quality of life) outcomes, [8,9] and are also cost-effective.[10] Therefore, DSME programs are recommended by most clinical guidelines.[7] However, there is considerable heterogeneity in the effectiveness of DSME programmes[8,9]. Programmes that are more effective usually offer more than 10 hours of contact between trainers and patients, incorporate behavioural approaches and provide longer-term support mechanisms. However, providing intensive and sustained support has cost implications, resulting in ongoing efforts to identify more cost-efficient ways to deliver DSME, notably through use of lay health workers or peer educators, such as Thai village health volunteers (VHV).

Peers can support sustained changes in complex health behaviours by providing assistance in daily management, social and emotional support, linkage to clinical care, and ongoing availability of support.[11,12] Unlike the educational/psychological framework of professional support, peer support operates on a social support framework. Although traditionally restricted to those with experience of disease, the definition of peers has been expanded to include other non-professionals with a close relationship with the community (e.g. VHV).[13] However, despite widespread interest, empirical data on effectiveness of peers in supporting behaviour change in chronic diseases, including diabetes, is limited and inconsistent.[11,12] In an earlier review, the World Health Organisation did not find sufficient evidence to recommend peer support programs as a policy option for diabetes management in LMICs.[14] Whereas many studies on the effectiveness of DSME programmes come from high-income countries (HIC), there is a

For peer review only

For peer review only - http://bmjopen.bmj.com/site/about/guidelines.xhtml

dearth of data from LMIC settings on cost-effectiveness, acceptability and potential adverse consequences of peer support programmes, as well as optimal strategies for mobilising and integrating peers in diabetes care pathways[12,17-18].

In the Thai healthcare system, structured DSME is not routinely available. While several small-scale studies from Thailand have demonstrated that DSME can strengthen self-management of diabetes, negative perceptions of educational programs and concerns about the burden on existing staff time and costs, have so far prevented the introduction of DSME.[1,18] However, recent policy developments in Thailand are supportive of DSME introduction, if a scalable model can be found. We therefore hypothesise that a nurse-led and/or peer-assisted model for DSME delivery will be effective in improving blood glucose among people with diabetes, with the peer-assisted model being the more scalable option for the Thai healthcare system. We propose to evaluate this through a 3-arm cluster randomised controlled trial.

METHODS AND ANALYSIS

Study design

This study is an MRC complex intervention[19] 3-arm cluster randomised controlled trial. Primary care units will be randomised for patients to receiving either the nurse-led or peer-assisted DSME intervention or standard care (brief education session by a nurse). Assessments will be undertaken at baseline, 6- and 12-month follow up. A process and cost-effective evaluation will also be conducted.

Randomisation

For peer review only - http://bmjopen.bmj.com/site/about/guidelines.xhtml

Twenty-one primary care units will be randomised to provide one of three interventions: (1) nurse-led DSME; (2) nurse-led DSME with peer assistance (provided by Thai village health volunteers, VHVs); or (3) standard care (brief education session by a nurse), resulting in seven primary care units in each arm of the study. All primary care units follow protocols for diabetes management as outlined by national guidelines. Randomisation will minimise any variation in practice between the different primary care units.

Sample size calculation

The intervention is powered to detect a clinically important difference of 0.6 units HbA1c (SD 1.5 units) between the control and intervention arms. Therefore, 693 participants are needed from 21 primary care units (7 in each trial arm arm) to achieve 80% power at 5% significance level, assuming an intra-cluster correlation coefficient (ICC) between primary care units of 0.02 and loss to follow-up rate of 20% [20,21].

Participant Selection

693 patients requiring a DSME intervention will be recruited from 21 primary care units in Chiang Mai and Lampang provinces in northern Thailand. While diabetes is diagnosed at tertiary hospitals, it is managed at the primary care unit health centres, which are served by a full-time nurse (doctor visits weekly), and 10-15 village health volunteers (VHV) linking patients in the community.

We will recruit all new referrals for diabetes management and patients with uncontrolled diabetes diagnosed in the past 3 years at the 21 primary care units over a 9-month period

For peer review only

(N=693). Posters and information sheets will be used to provide necessary, trial-related information to prospective participants.

Participants presenting to one of the 21 primary care units will be included if they are:

1. Over 18 years of age with a new referral for type 2 diabetes management;
2. Over 18 years of age with uncontrolled in diabetes (HbA1c>7 mg/dl) within the first three years of diagnosis;
2. Willing and able to attend educational group meetings; and
3. Available for six and 12-month follow-up visits

Participants will be excluded if they:

1. Have advanced diabetic complications such as diabetic nephropathy, diabetic retinopathy, or amputations; or if they are pregnant;
2. Have learning disabilities, dementia or active severe mental illness; or
3. Lack the capacity to give voluntary, informed consent.

Informed consent

Written informed consent will be obtained from all study participants in Thai before any study procedures are undertaken including enrolment, intervention allocation, follow-up interviews and blood draws. Local research assistants will explain the study to patients using the patient information sheet. The right of the patient to refuse to participate without giving reasons will be respected.

For peer review only

Figure 1: Trial flow diagram

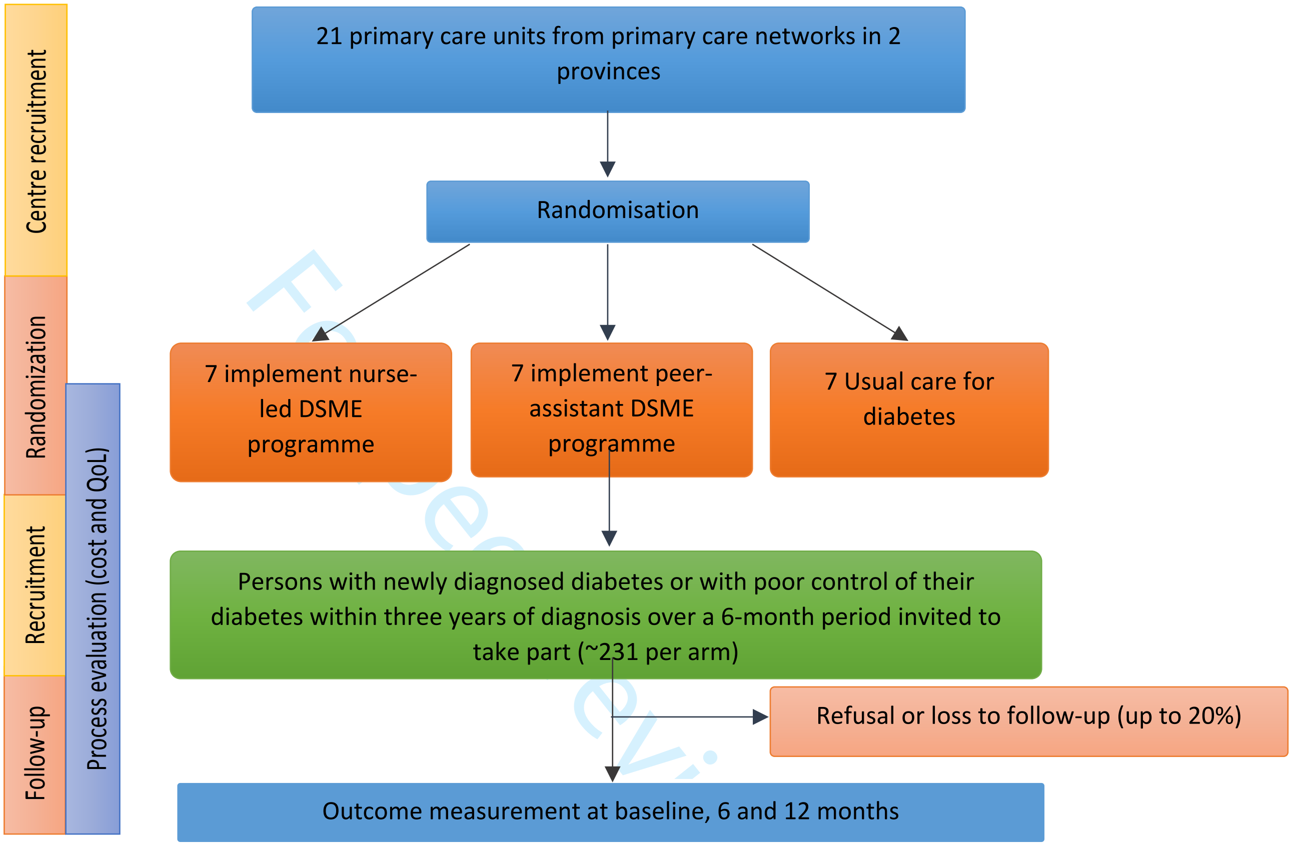

Intervention description and delivery

The DSME programme has been developed using a structured process. This included a desk and literature review and focus groups with local nurses and village health volunteers to develop a paper prototype of the intervention, including a hypothesised pathway of action (e.g. common-sense model of illness, empowerment, discovery learning, social learning, and social support) and a training manual for nurses and VHVs. In addition, seven brief films (5-6 minutes long) have been developed and have been used to trigger discussions on key topic areas during DSME intervention meetings covering such topics as medical adherence, dietary recommendation,

For peer review only - http://bmjopen.bmj.com/site/about/guidelines.xhtml

physical activity and stress management. Films will be used in the intervention as they are increasingly recognised as a highly effective medium for improved recall when communicating large amounts of information, particularly in low literacy settings[22]. Films will be in the local language and use local people.

The nurses and VHVs in the intervention arms will be trained to deliver the DSME programme (at the community hospital or neighbourhoods as appropriate). The intervention will be piloted at four community hospitals with four nurses and four VHVs who will be trained to deliver the DSME programme to groups of 5-10 persons with diabetes. This will allow for refining the intervention, ensuring data collection can be completed as specified, and to check our assumptions and processes for the trial.

A process evaluation at the end of the study will aim to assess intervention delivery (fidelity, dose and reach), clarify causal mechanisms (those hypothesised by theory of change developed within the project or identify unexpected ones), and identify contextual factors (barriers, facilitators) associated with variation in outcomes.[23] The process evaluation will consist of one-to-one interviews with clinicians and policy makers and direct observations of patients. Data for economic evaluation (resource usage and quality of life using EQ-5D[24]) will be obtained prospectively alongside the trial.

Standard care

Patients in the control group will receive standard care in the form a brief didactic educational session at the time of diagnosis of diabetes[25].

For peer review only - http://bmjopen.bmj.com/site/about/guidelines.xhtml

Study outcomes

The primary trial outcome of the intervention is a difference in trial arms at one-year follow-up in HbA1c. There is a growing recognition of the importance of combining tight glycaemic control with reduction in other cardiovascular risk factors for prevention of or reduction in complications.[4]

The cardiovascular risk will be estimated by the Thai cardiovascular risk score model, which estimates the risk of dying from any cardiovascular disease over 10 years based on age, gender, smoking habits, total cholesterol and systolic blood pressure, as it has been calibrated for use in a Thai population.[26] Additional measures include biophysical data, psychosocial and lifestyle data and intervention related data, as described in Table 1.

Table 1: Primary and Secondary Outcome measures

**Target domain**	**Measures or questionnaires**
**Cardiovascular risk assessment**	Haemoglobin A1c levels (HbA1c): HbA1c will measure the average blood glucose (sugar) levels months
	Blood lipids
	Body Mass Index; BMI
	Waist circumference
	Blood pressure
**Quality of life**	WHOQOL BREF- A 26 item questionnaire developed by WHO to assess quality of life in adults[27]
	The European Quality of Life questionnaire; EuroQol EQ-5D 5L[24]

For peer review only - http://bmjopen.bmj.com/site/about/guidelines.xhtml

	EQ-5D is a quality of life measure that includes five quality of life questions on mobility, self-care, usual activity, pain, anxiety / depression and a scale of 0 to 100 on how the person is feeling on that day.
**Depression**	Hospital Anxiety and Depression Scale; HADS[28]<br>HADS measures depression and anxiety that will address psychological change with a scale from 0 to 3.
**Stress**	Perceived stress questionnaire; PSS[29]<br>PSS is a psychological instrument for measuring the perception of stress. Ten items with a scale from 1 to 4.
**Physical activity**	International Physical Activity Questionnaire; IPAQ[30]<br>Short form IPAQ is an assessment of physical activity comprising of seven questions. There are two forms of output from scoring the IPAQ. Results can be reported in categories (low activity levels, moderate activity levels or high activity levels) or as a continuous variable (MET minutes a week). MET minutes represent the amount of energy expended carrying out physical activity.
**Diabetes knowledge**	Brief diabetes illness perception questionnaire; B-IPQ[31].

For peer review only - http://bmjopen.bmj.com/site/about/guidelines.xhtml

	B-IPQ has nine components of which the first five questions assess the cognitive representation of illness perception, two of the questions assess the emotional representation, one item assesses comprehensibility and one item on the root cause of the illness
	Diabetes Self-Management Education and Support; DMSES[32]. DMSES is one of the most widely used scale in measuring self-efficacy in type 2 diabetes management. The Thai-DMSES has twenty questions which has been demonstrated to have good psychometric properties[32]
	Summary of Diabetes self-care activities questionnaire SDSCA[34] SDSCA is a diabetes self-care activities questionnaire focusing on general diet, diabetes-specific diet, physical activity, blood-glucose testing, foot care, and smoking.
**Satisfaction with intervention**	Chronic Illness Resources survey (CIRS)[35] CIRS is a questionnaire to represent patient's received support. Individual's support for behavioural-specific disease management is assessed: proximal support e.g. friend and family and distal factor e.g. neighbourhood or community.
	Modified Medical Interview Satisfaction scale (MISS-21)[36] MISS-21 is a questionnaire to measure patient satisfaction with patient and health care professional communication/consultation.

For peer review only

Data collection and follow-up

Each participant will be involved in the study for 12 months after taking consent and baseline data. The trial is expected to start January 2020 and finish October 2021.

Data collection methods will include:

a) Questionnaires: Questionnaire data will be collected face-to-face by research assistants for the full sample at baseline, 6 and 12 months at the community hospital where participants are recruited (Table 1). A custom-designed form linked to Microsoft Access will be used to collect, validate, verify, and store respondents' data where possible or else data will be collected via paper forms and double-entered into the databases. All data files and databases will be password protected.

b) Biological samples: Blood samples will be collected at baseline, 6 and 12 months, to measure fasting blood glucose, HbA1c and lipids, coordinating where possible with the annual routine tests offered to patients to reduce duplication. All blood samples will be administered from participants by trained phlebotomists. Data will be linked to the participant information using a unique respondent ID, which will be assigned to all study participants.

c) Interviews: During the delivery of the intervention, a process evaluation using a subset of participants will be followed up using in-depth interviews and focus group discussions. These will be audio-recorded. Data will be collected using a range of qualitative methods: a) one to one interviews, and focus group discussions with nurses, health volunteers, people with diabetes and their carers (5-10 focus groups, and 20 semi-structured interviews) and b) ethnography

For peer review only - http://bmjopen.bmj.com/site/about/guidelines.xhtml

through direct observations including video recordings of intervention delivery, and unstructured interviews with clinical managers and policy makers. The data collected will be used to capture the range of experiences of the intervention, and identify unanticipated pathways to generate new theories as well as exploring the scalability of the intervention.

Trial follow-up appointments

The research team will hold weekly briefings with the study coordinators to generate a list of priority areas and loss to follow-up participant lists. Arrangements to follow-up participants who have not turned up for their appointment will be made, with attempts to contact participants through SMS, phone calls, or house visits. Participants will be declared loss to follow-up if they do not show for a month and are untraceable.

Data management

A data collection protocol will be developed, and the study coordinator in Thailand will provide training to fieldworkers before data collection commences. Validation will be performed a random sample of questionnaire data by crosschecking with clinic records. Any discrepancies will be followed-up and addressed by field workers, re-contacting participants to clarify as necessary. Quantitative data will be entered directly via a form with built-in data checks to minimise transcription errors (or where necessary collected on paper and later double entered into the electronic form). Post entry checks will be conducted using statistical software. All hospital laboratories have their own internal quality assurance protocols and are also linked to a national external quality assurance mechanism. Fieldworkers will be trained in qualitative methods and an interview schedule will be devised.

For peer review only - http://bmjopen.bmj.com/site/about/guidelines.xhtml

Statistical analysis

1. Quantitative analysis

Available outcome data will be analysed on an intention-to-treat basis. Potential clustering of outcomes at the level of community primary care units will be accounted for using mixed-effects models. Adjustment for baseline imbalances in outcomes or relevant covariates will be considered as appropriate.

2. Qualitative analysis

Qualitative data from interviews, focus groups and direct observations be will transcribed and analysed using NVivo software. The data will be analysed using a descriptive, phenomenological approach to understand participants' experiences and interpret them within their respective cultural contexts. Comparative analysis will compare and contrast these themes across participants. Deviant cases will be actively sought throughout the analysis and emerging ideas and themes modified in response. In addition, thematic analysis will be used to inform elements of scalability and to produce a set of considerations in making decisions about the scalability of the intervention.

ETHICS AND DISSEMINATION

This study is to be conducted according to the international standards of Good Clinical Practice (International Conference on Harmonization guidelines), Declaration of Helsinki, and International Ethical Guidelines for Biomedical Research Involving Human Subjects, applicable national government regulations, and institutional research policies and procedures. All

For peer review only

investigators will receive GCP training at the onset of the study. Ethical approvals will be sought prior to commencement of the project from Thailand's Central Research Ethics Committee and the London School of Hygiene & Tropical Medicine. The study protocol, informed consent form, participant's information sheet and other relevant information has been submitted to and approved by Chiang Mai University and local Ethics Committee. Any future amendments of the protocol shall be submitted to and approved by the Institutional Review Board (IRB) before implementation

Trial monitoring and oversight

The role of the Trial Steering Committee (TSC) is to provide overall supervision of the trial. The TSC will meet every six months. The TSC will include experts in the field of DSME, health psychology and clinical trials, as well as an independent Chair. In addition, we will have patient and carer representatives and policy representation.

Dissemination

Expected output and impact research findings will be disseminated to scientific audiences at major conferences and published in high-impact open-access scientific journals; planned publications include those on intervention development, primary trial results, and process evaluation, and health systems analysis, at a minimum. This study is expected to have a major policy impact due to the close involvement of a key policy maker in the project. Towards the end of the study a dedicated workshop will be held with key governmental stakeholders to disseminate the recommended model for DSME implementation in Thailand and encourage inclusion of a large-scale scientific evaluation into any national implementation of the scheme.

For peer review only - http://bmjopen.bmj.com/site/about/guidelines.xhtml

Contributors: CA, SS, OQ, PM, SK were involved in conception and trial design. CA, KP, PM and SK wrote the first draft of the trial design proposal. KR, WT, KH, KK were involved in critical revision of the trial design proposal. All authors contributed to finalising the trial protocol. CA, KP, IP, CP, AH, NW were involved with preparation of the manuscript for submission. All authors reviewed the manuscript and approved the manuscript for publication.

Funding

This study is supported by UK Medical Research Council (MRC) grant number [MR/R020876/1] and the Thailand Research Fund (TRF) grant number [DBG6180007].

Data availability

Data will be shared upon reasonable request

Patient and Public Involvement

Patients or the public WERE NOT involved in the design, or conduct, or reporting, or dissemination plans of our research

Trial sponsor

This study is sponsored by London School of Hygiene & Tropical Medicine, Keppel Street, London WC1E 7HT, UK [16113].

Competing interests

None declared

For peer review only

Ethics approval

The study has been approved by the Chiang Mai University Research Ethics Committee [326/2018] and the London School of Hygiene & Tropical Medicine [16113/RR/12850].

REFERENCES

1. Deerochanawong C, Ferrario A. Diabetes management in Thailand: a literature review of the burden, costs, and outcomes. Glob Health 2013; 9: 11.
2. International Diabetes Federation. IDF Diabetes Atlas Seventh Edition. 2015.
3. Effect of intensive blood-glucose control with metformin on complications in overweight patients with type 2 diabetes (UKPDS 34). The Lancet 1998; 352: 854–65.
4. Gaede P, Vedel P, Larsen N, Jensen GVH, Parving H-H, Pedersen O. Multifactorial intervention and cardiovascular disease in patients with type 2 diabetes. N Engl J Med 2003; 348: 383–93.
5. Rangsin R. An assessment of quality of care among patients diagnosed with type 2 diabetes and hypertension visiting hospitals of ministry of public health and Bangkok Metropolitan Administration in Thailand. 2012.
6. Auemaneekul N, Chandanasotthi P, Rawiworrakul T, et al. Community Partnership, Contextbased Intervention and Diabetes Control in Thailand. J Public Health 2016; 46: 236–47.
7. Haas L, Maryniuk M, Beck J, et al. National standards for diabetes self-management education and support. Diabetes Educ 2012; 38: 619–629.

For peer review only - http://bmjopen.bmj.com/site/about/guidelines.xhtml

8. Chrvala CA, et al. Diabetes self-management education for adults with type 2 diabetes mellitus: A systematic review of the effect on glycemic control. Patient Educ Couns 2016; 99: 926–43.

9. Pillay J, Armstrong MJ, Butalia S, et al. Behavioral Programmes for Type 2 Diabetes Mellitus: A Systematic Review and Network Meta-analysis. Ann Intern Med 2015; 163: 848.

10. Doyle G, O'Donnell S, Quigley E, et al. Patient level cost of diabetes self-management education programmes: an international evaluation. BMJ Open 2017; 7: e013805.

11. Fisher EB, Boothroyd RI, Elstad EA, et al. Peer support of complex health behaviors in prevention and disease management with special reference to diabetes: systematic reviews. Clin Diabetes Endocrinol 2017; 3. DOI:10.1186/s40842-017-0042-3.

12. Dale JR, Williams SM, Bowyer V. What is the effect of peer support on diabetes outcomes in adults? A systematic review. Diabet Med 2012; 29: 1361–77.

13. Norris SL, Chowdhury FM, Van Le K, et al. Effectiveness of community health workers in the care of persons with diabetes. Diabet Med J Br Diabet Assoc 2006; 23: 544–56.

14. World Health Organization. Peer support programmes in diabetes: Report of a WHO consultation 5-7 November 2007. Geneva, Switzerland: WHO, 2008.

15. Fisher EB, Boothroyd RI, Coufal MM, et al. Peer Support For Self-Management Of Diabetes Improved Outcomes In International Settings. Health Aff (Millwood) 2012; 31: 130–9.

For peer review only - http://bmjopen.bmj.com/site/about/guidelines.xhtml

16. Trento M, Gamba S, Gentile L, et al. Rethink Organization to iMprove Education and Outcomes (ROMEO): A multicenter randomized trial of lifestyle intervention by group care to manage type 2 diabetes. Diabetes Care 2010; 33: 745–7.

17. Werfalli M, Raubenheimer P, Engel M, et al. Effectiveness of community-based peer-led diabetes self-management programmemes (COMP-DSMP) for improving clinical outcomes and quality of life of adults with diabetes in primary care settings in low and middle-income countries (LMIC): a systematic review and meta-analysis. BMJ Open 2015; 5: e007635.

18. Keeratiyutawong P, Hanucharurnkl S, Melkus G, Vorapongsathon OPT. Effectiveness of a self-management programme for Thais with type 2 diabetes. Thai J Nurs Res 2006; 10: 85–97.

19. Craig P, Dieppe P, Macintyre S, Michie S, Nazareth I, Petticrew M. Developing and evaluating complex interventions: the new Medical Research Council guidance. BMJ 2008; a1655.

20. Wattana C, Srisuphan W, Pothiban L, Upchurch SL. Effects of a diabetes self-management programme on glycaemic control, coronary heart disease risk, and quality of life among Thai patients with type 2 diabetes. Nurs Health Sci 2007; 9: 135–41.

21. Khunti K, Gray LJ, Skinner T, et al. Effectiveness of a diabetes education and self-management programme (DESMOND) for people with newly diagnosed type 2 diabetes mellitus: Three-year follow-up of a cluster randomised controlled trial in primary care. BMJ 2012; 344: e2333–e2333.

For peer review only - http://bmjopen.bmj.com/site/about/guidelines.xhtml

22. Tuong W, Larsen ER, Armstrong AW. Videos to influence: a systematic review of effectiveness of video-based education in modifying health behaviors. J Behav Med 2014; 37: 218–33.

23. Moore GF, Audrey S, Barker M, et al. Process evaluation of complex interventions: Medical Research Council guidance. BMJ 2015; 350: h1258–h1258. 25Mangham LJ, Hanson K. Scaling up in international health: what are the key issues? Health Policy Plan 2010; 25: 85–96.

24. Kimman M, et al. Validity of the Thai EQ-5D in an occupational population in Thailand. *Qual Life Res* 2013 Aug; 22(6):1499-506.

25. Clinical Practice Guidelines for Diabetes. Diabetes Association of Thailand, 2017. (https://www.dmthai.org/index.php/knowledge/healthcare-providers/cpg/443-guideline-diabetes-care-2017)

26. Aekplakorn A, Bunnag P, Woodward M. et al. Risk Score for Predicting Incident Diabetes in the Thai Population. *Diabetes Care* 2006 Aug; 29(8):1872-1877

27. Mahatnirunkul S. et al. Comparison of the WHOQOL-100 and the WHOQOL-BREF. *Journal of Mental Health of Thailand* 1998; 5(3).

28. Nilchaikovit T. et al. Development of Thai version of Hospital Anxiety and Depression Scale in cancer patients. *J Psychiatr Assoc Thailand* 1996; 41(1):18-30.

29. Wongpakaran N, Wongpakaran T. The Thai version of the PSS-10: An investigation of its psychometric properties. *BioPsychoSocial Medicine* 2010: 4:6

30. Rattanawiwatpong P, et al. Validity and reliability of the Thai version of Short Format International Physical Activity Questionnaire (IPAQ). *J Thai Rehabil* 2006; 16(3):147-160.

For peer review only - http://bmjopen.bmj.com/site/about/guidelines.xhtml

31. Leelacharas S, et al. Illness perceptions, lifestyle behaviours, social support and cardiovascular risks in people with Hypertension in Urban and Rural Areas of Thailand. Pacific Rim Int J Nur Res 2015; 19

32. Lamsumang, W. The development of the Thai version of the diabetes management self-efficacy scale (T-DMSES) for older adults with type 2 diabetes. State University of New York at Buffalo, New York; 2009.

33. Sangruangake M, Jirapornkul C, Hurst C. Psychometric Properties of Diabetes Management Self-Efficacy in Thai Type 2 Diabetes Mellitus Patients: A Multicenter Study. *Int J Endocrinol*. 2017;2017:2503156. doi:10.1155/2017/2503156)

34. Keeratiyatawong, P, Hanucharumkul, S., Melkus, G. D. E., Panpakdee, O., Vorapongsathorn T. Effectiveness of a self-management programme for Thais with type 2 diabetes. Thai J Nurse Res. 2006; 10: 85-97

35. Manit A, et al. Development of needs and resources for self-management assessment instrument in Thais with type 2 diabetes: Cross-cultural adaptation. *J Med Assoc Thai* 2011; Vol. 94 No. 11.

36. Meakin R and Weinman J. The 'Medical Interview Satisfaction Scale' (MISS-21) adapted for British general practice. *Family Practice* 2002; 19:257–263.

For peer review only

For peer review only - http://bmjopen.bmj.com/site/about/guidelines.xhtml

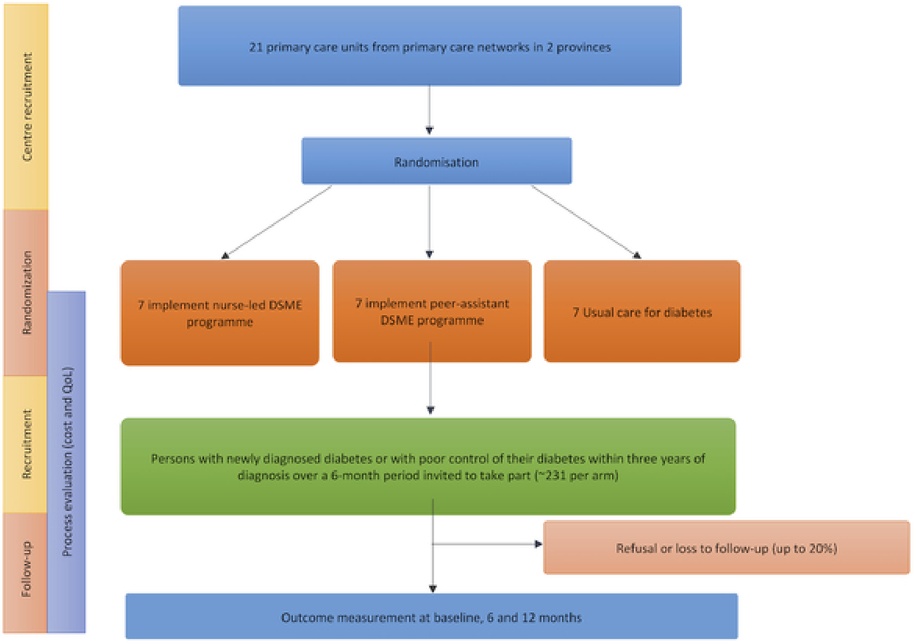

Figure 1: Trial flow diagram

27x20mm (600 x 600 DPI)

BMJ Open

A Scalable Solution for Delivery of Diabetes Self-Management Education in Thailand (DSME-T): A Cluster Randomized Trial Study Protocol

Journal:	*BMJ Open*
Manuscript ID	bmjopen-2020-036963.R1
Article Type:	Protocol
Date Submitted by the Author:	28-Jun-2020
Complete List of Authors:	Angkurawaranon, Chaisiri; Chiang Mai University, Department of Family Medicine, Faculty of Medicine<br>Papachristou Nadal, Iliatha; London School of Hygiene and Tropical Medicine, Faculty of Epidemiology and Population Health<br>Mallinson, Poppy; London School of Hygiene and Tropical Medicine Faculty of Epidemiology and Population Health, Department of Non-communicable Disease Epidemiology<br>Pinyopornpanish, Kanokporn; Chiang Mai University, Department of Family Medicine, Faculty of Medicine<br>Quansri, Orawan; Mahidol University<br>Rerkasem, Kittipan ; Chiang Mai University, Department of Surgery, Faculty of Medicine, Chiang Mai University; Chiang Mai University, Research Institute for Health Sciences<br>Srivanichakorn, Supattra; Royal Thai Government Ministry of Public Health<br>Techakehakij, Win; Lampang Hospital<br>Wichit, Nutchanath; Surat Thani Rajabhat University<br>Pateekhum, Chanapat; Chiang Mai University<br>Hashmi, Ahmar; Chiang Mai University, Department of Family Medicine<br>Hanson, Kara; London School of Hygiene and Tropical Medicine<br>Khunti, Kamlesh; University of Leicester, Department of Health Sciences<br>Kinra, Sanjay; London School of Hygiene and Tropical Medicine
**Primary Subject Heading**:	Diabetes and endocrinology
Secondary Subject Heading:	Evidence based practice, Diabetes and endocrinology, General practice / Family practice
Keywords:	DIABETES & ENDOCRINOLOGY, EDUCATION & TRAINING (see Medical Education & Training), Protocols & guidelines < HEALTH SERVICES ADMINISTRATION & MANAGEMENT

SCHOLARONE™
Manuscripts

I, the Submitting Author has the right to grant and does grant on behalf of all authors of the Work (as defined in the below author licence), an exclusive licence and/or a non-exclusive licence for contributions from authors who are: i) UK Crown employees; ii) where BMJ has agreed a CC-BY licence shall apply, and/or iii) in accordance with the terms applicable for US Federal Government officers or employees acting as part of their official duties; on a worldwide, perpetual, irrevocable, royalty-free basis to BMJ Publishing Group Ltd ("BMJ") its licensees and where the relevant Journal is co-owned by BMJ to the co-owners of the Journal, to publish the Work in this journal and any other BMJ products and to exploit all rights, as set out in our licence.

The Submitting Author accepts and understands that any supply made under these terms is made by BMJ to the Submitting Author unless you are acting as an employee on behalf of your employer or a postgraduate student of an affiliated institution which is paying any applicable article publishing charge ("APC") for Open Access articles. Where the Submitting Author wishes to make the Work available on an Open Access basis (and intends to pay the relevant APC), the terms of reuse of such Open Access shall be governed by a Creative Commons licence – details of these licences and which Creative Commons licence will apply to this Work are set out in our licence referred to above.

Other than as permitted in any relevant BMJ Author's Self Archiving Policies, I confirm this Work has not been accepted for publication elsewhere, is not being considered for publication elsewhere and does not duplicate material already published. I confirm all authors consent to publication of this Work and authorise the granting of this licence.

For peer review only

A Scalable Solution for Delivery of Diabetes Self-Management Education in Thailand (DSME-T): A Cluster Randomized Trial Study Protocol

Chaisiri Angkurawaranon[1*], Iliatha Papachristou Nadal[2,3], Poppy Mallinson[2,] Kanokporn Pinyopornpanish[1], Orawan Quansri[4], Kittipan Rerkasem[5,6], Supattra Srivanichakorn[7], Win Techakehakij[8], Nutchanath Wichit[9], Chanapat Pateekhum[1], Ahmar Hashmi[1], Kara Hanson[10], Kamlesh Khunti[11], Sanjay Kinra[2]

Author affiliations

[1] Department of Family Medicine, Faculty of Medicine, Chiang Mai University

[2] Faculty of Epidemiology and Population Health, London School of Hygiene and Tropical Medicine

[3] Psychological Medicines, King's College London

[4] ASEAN Institute for Health Development, Mahidol University

[5] Department of Surgery, Faculty of Medicine, Chiang Mai University

[6] NCD Centre of Excellence, Research Institute for Health Sciences, Chiang Mai University

[7] Department of Disease Control, Ministry of Public Health

[8] Department of Social Medicine, Lampang Hospital

[9] Faculty of Nursing, Suratthanni Rajabhat University

[10] Faculty of Public Health and Policy, London School of Hygiene and Tropical Medicine

[11] Diabetes Research Center, University of Leicester

Corresponding author: Chaisiri Angkurawaranon, Department of Family Medicine, Faculty of Medicine, Chiang Mai University, email: chaisiri.a@cmu.ac.th

For peer review only - http://bmjopen.bmj.com/site/about/guidelines.xhtml

ABSTRACT

Introduction Type 2 diabetes mellitus is amongst the foremost health challenges facing policy makers in Thailand as its prevalence has more than tripled over the last two decades, accounting for considerable death, disability and healthcare expenditure. Diabetes Self-Management Education (DSME) programmes show promise in improving diabetes outcomes, but this is not routinely utilised in Thailand. This study aims to test a culturally-tailored DSME model in Thailand, using a 3-arm cluster randomised controlled trial comparing a nurse-led model, a peer-assisted model, and standard care. We will test which model is effective and cost effective to improve cardiovascular risk and control of blood glucose among people with diabetes.

Methods and analysis 21 primary care units in northern Thailand will be randomised to one of three interventions, enrolling a total of 693 patients. The primary care units will be randomised (1:1:1) to participate in a culturally-tailored DSME intervention for 12 months. The three-arm trial design will compare effectiveness of nurse-led, peer-assisted (Thai village health volunteers) and standard care. The primary trial outcomes are changes in haemoglobin A1c and cardiovascular risk score. A process evaluation and cost effectiveness evaluation will be conducted to produce policy relevant guidance for the Thai Ministry of Public Health. The planned trial period will start in January 2020 and finish October 2021.

Ethics and dissemination Ethical approval has been obtained from Thailand and the UK. We will share our study data with other researchers, advertising via our publications and web presence. In particular, we are committed to sharing our findings and data with academic audiences in Thailand and other low- and middle-income countries.

For peer review only - http://bmjopen.bmj.com/site/about/guidelines.xhtml

Trial registration number:

ClinicalTrials.gov ID NCT03938233

Strengths and limitation of this study

- A three-arm cluster randomized control trial to evaluate clinical and cost-effectiveness of a culturally tailored DSME under two alternative modes of delivery (nurse-led and peer-assisted) will provide policy makers with options for scalability.
- A culturally-tailored DSME programme has been developed with input from stakeholders (policy makers, clinicians, nurses, village health volunteers and people with diabetes).
- A series of short films has been developed to introduce key topics, as there is increasing recognition that films are a highly efficient medium for communicating information, particularly in low literacy settings

For peer review only - http://bmjopen.bmj.com/site/about/guidelines.xhtml

INTRODUCTION

Type 2 diabetes mellitus (hereto referred to as diabetes) is amongst the foremost health challenges facing policy makers in Thailand. Its prevalence has more than tripled over the last two decades to an estimated 4 million adults (age adjusted prevalence 7.1%) living with diabetes in 2015.[1,2] Diabetes is associated with several macrovascular (e.g. ischaemic heart disease) and microvascular complications (e.g. nephropathy, retinopathy, neuropathy, and foot disease), which primarily account for the considerable death and disability (of which diabetes is the 5th leading cause in Thailand). In addition, diabetes in Thailand causes two-fold increase in healthcare expenditure and significant loss of economic productivity—of both people with diabetes and their carers.[1–4]

The complications of diabetes can be largely prevented or delayed through lifestyle change and medication when necessary, and regular screening for early detection and management of complications to control risk factors such as blood glucose, lipids and blood pressure.[3,4] Under Thailand's universal health coverage, nearly everyone diagnosed with diabetes receives timely medical care (>97%) and has access to screening. Yet, surveys suggest that only about half of the people with diabetes achieve optimal control of risk factors or receive annual screening for microvascular complications (53-60%).[1,5] Limited data support a lack of engagement and self-management skills among those diagnosed with diabetes as the main underlying reasons for this.[6]
Successful management of diabetes involves a considerable degree of self-management. People with diabetes need to adhere to multiple behaviours, including healthy lifestyles, regular monitoring and medication, problem-solving and healthy coping strategies. In this, they are greatly supported by diabetes self-management education (DSME), defined as 'a collaborative and ongoing process intended to facilitate the development of knowledge, skills, and abilities that are required for successful self-management of diabetes'.[7] Evidence from over 100 studies,

For peer review only

including many randomised controlled trials conducted predominantly in high-income countries, suggests that DSME programmes are associated with improvements in a range of behavioural outcomes (knowledge, behaviours, self-efficacy, psychosocial), and clinical outcomes (physiological risk factors, screening for complications, quality of life), [8,9] and are-cost-effective.[10] Therefore, DSME programmes are recommended by most clinical guidelines.[7] However, there is considerable heterogeneity in the effectiveness of DSME programmes[8,9]. Programmes that are more effective usually offer more than 10 hours of contact between trainers and patients, incorporate behavioural approaches and provide longer-term support mechanisms. However, providing intensive and sustained support has cost implications, resulting in ongoing efforts to identify more cost-efficient ways to deliver DSME, notably through use of lay health workers or peer educators, such as Thai village health volunteers (VHV).

Peers can support sustained changes in complex health behaviours by providing assistance in daily management, social and emotional support, linkage to clinical care, and ongoing availability of support.[11,12] Unlike the educational/psychological framework of professional support, peer support operates on a social support framework. Although traditionally restricted to those with experience of disease, the definition of peers has been expanded to include other non-professionals with a close relationship with the community (e.g. VHV).[13] However, despite widespread interest, empirical data on effectiveness of peers in supporting behaviour change in chronic diseases, including diabetes, is limited and inconsistent.[14,15] In an earlier review, the World Health Organisation did not find sufficient evidence to recommend peer support programmes as a policy option for diabetes management in LMICs.[16] Whereas many studies on the effectiveness of DSME programmes come from high-income countries (HIC), there is a dearth of data from LMIC settings on cost-effectiveness, acceptability and potential adverse

For peer review only - http://bmjopen.bmj.com/site/about/guidelines.xhtml

consequences of peer support programmes, as well as optimal strategies for mobilising and integrating peers in diabetes care pathways.[12,17-18]

In the Thai healthcare system, structured DSME is not routinely available. While several small-scale studies from Thailand have demonstrated that DSME can strengthen self-management of diabetes, negative perceptions of educational programmes and concerns about the burden on existing staff time and costs, have so far prevented the introduction of DSME.[1,18] However, recent policy developments in Thailand are supportive of DSME introduction, if a scalable model can be found. We therefore hypothesise that a nurse-led and/or peer-assisted model for DSME delivery will be effective in improving blood glucose among people with diabetes, with the peer-assisted model being the more scalable option for the Thai healthcare system. We propose to evaluate this through a three-arm cluster randomised controlled trial.

METHODS AND ANALYSIS

Study design

This study is a MRC complex intervention,[19] three-arm cluster randomised controlled trial. Primary care units from within two provinces: Chiang Mai and Lampang will be randomised for patients to receive either the nurse-led or peer-assisted DSME intervention or standard care (brief education session by a nurse). Assessments will be undertaken at baseline, 6- and 12-month follow up. A process and cost-effective evaluation will also be conducted.

Setting and Participant Selection

Potential participants requiring a DSME intervention will be recruited from 21 primary care units in Chiang Mai (7 primary care units) and Lampang provinces (14 primary care units) in northern

For peer review only - http://bmjopen.bmj.com/site/about/guidelines.xhtml

Thailand. Chiang Mai is a province of over 1.4 million people with 24 district hospitals and about 250 primary care units. Lampang is a province of approximately 700,000 people with 12 district hospitals and about 140 primary care units. While diabetes is diagnosed at tertiary and district hospitals, it is managed at the primary care unit health centres, which are served by a full-time nurse (doctor visits weekly), and 10-15 village health volunteers (VHV) linking patients in the community.

From clinical records, we will recruit all new referrals for diabetes management and patients with uncontrolled diabetes diagnosed in the past 3 years at the 21 primary care units over a 9-month period (N=693). Posters and information sheets will be used to provide necessary, trial-related information to prospective participants (Figure 1).

Participants presenting to one of the 21 primary care units will be included if they are:

1. Over 18 years of age with a new referral for type 2 diabetes management;
2. Over 18 years of age with uncontrolled diabetes (HbA1c>7 %) within the first three years of diagnosis;
2. Willing and able to attend educational group meetings; and
3. Available for 6- and 12-month follow-up visits

Participants will be excluded if they:

1. Have advanced diabetic complications such as diabetic nephropathy, diabetic retinopathy, or amputations; or if they are pregnant;
2. Have learning disabilities, dementia or active severe mental illness; or
3. Lack the capacity to give voluntary, informed consent.

For peer review only

For peer review only - http://bmjopen.bmj.com/site/about/guidelines.xhtml

Randomisation

Stratified by province, 21 primary care units (7 from Chiang Mai and 14 from Lampang) will be randomised to provide one of three interventions: (1) nurse-led DSME; (2) nurse-led DSME with peer assistance (provided by Thai village health volunteers, VHV); or (3) standard care (brief education session by a nurse), resulting in seven primary care units in each arm of the study. All primary care units follow protocols for diabetes management as outlined by national guidelines. Randomisation by province will minimise any variation in practice between the different primary care units.

Sample size calculation

The trial is powered to detect a difference in HbA1c of 0.6% (SD 1.5%) between control and intervention arms, based on the effect size of 0.6% noted in a previous diabetes management study in Thailand[20], and the fact that an increase in HbA1c of ~0.5% was associated with increased mortality among people with diabetes[21]. An ICC between hospitals of 0.02 was assumed based on a similar study which found that The intraclass correlation for HbA1c at three years was 0.02 (95% confidence interval 0.00 to 0.08)[22]. Allowing for a loss to follow up rate of 20%, 693 participants are needed from 21 primary care units (7 in each trial arm arm) to achieve 80% power at 2.5% significance level.

Informed consent

Written informed consent will be obtained from all study participants in Thai before any study procedures are undertaken including enrolment, intervention allocation, follow-up interviews and blood draws. Local research assistants will explain the study to patients using the patient

For peer review only - http://bmjopen.bmj.com/site/about/guidelines.xhtml
For peer review only

information sheet (supplementary file). The right of the patient to refuse to participate without giving reasons will be respected.

Intervention description and delivery

The DSME programme has been developed using a structured process. This included a desk and literature review and focus groups with local nurses and VHV to develop a paper prototype of the intervention, including a hypothesised pathway of action (e.g. common-sense model of illness, empowerment, discovery learning, social learning, and social support) and a training manual for nurses and VHV. In addition, seven brief films (5-6 minutes long) have been developed and have been used to trigger discussions on key topic areas during DSME intervention meetings covering such topics as medical adherence, dietary recommendation, physical activity and stress management. Films will be used in the intervention as they are increasingly recognised as a highly effective medium for improved recall when communicating large amounts of information, particularly in low literacy settings.[23] Films will be in the local language and use local people.

Our DSME programme will consist of 4 modules. Module 1 covers the general overview of diabetes, treatment targets and goal settings. Module 2 covers diet and nutrition. Module 3 covers physical activity and exercise while module 4 covers stress management and mental health. Each module takes approximately 1.5 hours. Each participant is given an information and self-assessment booklet which covers all contents and materials for the four modules.

In addition to routine care, in the nurse-led arm, the nurse will deliver the DSME to groups of 5-10 participants per session within the first months after enrolment. The participants in the nurse-

For peer review only - http://bmjopen.bmj.com/site/about/guidelines.xhtml

led arm will also be given a refresher session going over all 4 modules again at 6 months after enrolment. For the peer-assisted arm, a peer volunteer will participate as an assistant to the nurse in the first DSME session. However, the peer will lead the refresher course at 6 months. In addition, participants in the peer-led arm will received monthly contact with the peer either via a home visit or telephone call. During these brief 15-20 minute monthly contacts, peers will ask about the progress made, providing encouragement if plans for self-management are being followed or discussing ways to overcome barriers and set new goals if obstacles are identified. (Table 1)

For peer review only

Table 1 Summary DSME Delivery

Month	Routine care	Nurse-led DSME	Peer-assisted DSME**
0	Individual session	Nurse provides DSME (4 modules)	Nurse provides DSME (4 modules) with peer volunteers to assist the sessions
6	Individual session	Refresher course (4 modules) provided by nurse	Refresher course (4 modules) led by peers
12	Outcome assessment	Outcome assessment	Outcome assessment

* participants in the peer-led arm will received monthly contact with the peer either via a home visit or telephone call.

The intervention will be piloted at four community hospitals with four nurses and four VHV who will be trained to deliver the DSME programme to groups of 5-10 persons with diabetes. This will allow for refining the intervention, ensuring data collection can be completed as specified, and to check our assumptions and processes for the trial. For the main trial, a two-day workshop will be held such that at least one nurse and one VHV from each primary care unit will be trained by the Thai research team to deliver the DSME programme at the community hospital or neighbourhoods as appropriate. The trial coordinator will conduct periodic site visits as additional training as requested and a line of communication will be established between the research team and each site to answer any issues which may arise.

For peer review only

For peer review only - http://bmjopen.bmj.com/site/about/guidelines.xhtml

A process evaluation using qualitative methods will be conducted during the trial period and at the end of the study. Observations including video recordings of intervention delivery will be made. We plan to conduct 5-10 focus group among providers (nurses and village health volunteers) to explore healthcare professionals' perspectives regarding their experience and implementation of the DSME programme, including views on the cultural transferability of DSME and scalability to the Thai context. In addition, we planned to conduct 20 structured interviews with patients. These evaluations will help assess intervention delivery (fidelity, dose and reach), clarify causal mechanisms (those hypothesised by theory of change developed within the project or emergent mechanisms identified), and detail contextual factors (barriers, facilitators) associated with variation in outcomes.[24] The process evaluation will also consist of one-to-one interviews with clinicians and policy makers and direct observations of patients. Data for economic evaluations (resource usage and quality of life using EQ-5D[25]) will be obtained prospectively alongside the trial.

Standard care

Patients in the control group will receive standard care in the form of a brief didactic educational session at the time of diagnosis of diabetes.[26]

Study outcomes

The two primary outcomes of the intervention are a difference in trial arms at one-year follow-up in HbA1c and cardiovascular risk score. There is a growing recognition of the importance of combining tight glycaemic control with reduction in other cardiovascular risk factors for prevention of or reduction in complications.[4] The cardiovascular risk will be estimated by the

For peer review only - http://bmjopen.bmj.com/site/about/guidelines.xhtml

Thai cardiovascular risk score model, which estimates the risk of dying from any cardiovascular disease over 10 years based on age, gender, smoking habits, total cholesterol and systolic blood pressure, as it has been calibrated for use in a Thai population.[27]

Additional secondary outcomes include changes at one year for biophysical data, psychosocial and lifestyle data and intervention related data, as described in Table 2.

Table 2: Primary and secondary Outcome measures

**Primary outcomes**	**Measures or questionnaires**
**Haemoglobin A1c levels (HbA1c)**	HbA1c will measure the average blood glucose (sugar) levels over the past two to three months
**Thai Cardiovascular risk score**	Estimates the risk of dying from any cardiovascular disease over 10 years based on age, gender, smoking habits, total cholesterol and systolic blood pressure.
**Secondary outcomes**	**Measures or questionnaires**
**Biological and physical measures**	Body weight, Body Mass Index; BMI, Blood lipids (LDL-C), waist circumference
**Quality of life**	WHOQOL BREF- A 26 item questionnaire developed by WHO to assess quality of life in adults[28]
	The European Quality of Life questionnaire; EuroQol EQ-5D 5L[25] EQ-5D is a quality of life measure that includes five quality of life questions on mobility, self-care, usual activity, pain, anxiety/depression and a scale of 0 to 100 on how the person is feeling on that day.

For peer review only - http://bmjopen.bmj.com/site/about/guidelines.xhtml

**Depression**	Hospital Anxiety and Depression Scale; HADS[29]<br>HADS measures depression and anxiety that will address psychological change with a scale from 0 to 3.
**Stress**	Perceived stress questionnaire; PSS[30]<br>PSS is a psychological instrument for measuring the perception of stress. Ten items with a scale from 1 to 4.
**Physical activity**	International Physical Activity Questionnaire; IPAQ[31]<br>Short form IPAQ is an assessment of physical activity comprising of seven questions. There are two forms of output from scoring the IPAQ. Results can be reported in categories (low activity levels, moderate activity levels or high activity levels) or as a continuous variable (MET minutes a week). MET minutes represent the amount of energy expended carrying out physical activity.
**Diabetes knowledge and skills**	Brief diabetes illness perception questionnaire; B-IPQ[32].<br>B-IPQ has nine components of which the first five questions assess the cognitive representation of illness perception, two of the questions assess the emotional representation, one item assesses comprehensibility and one item on the root cause of the illness
	Diabetes Self-Management Education and Support; DMSES[33].<br>DMSES is one of the most widely used scale in measuring self-efficacy in type 2 diabetes management. The Thai-DMSES has twenty questions which has been demonstrated to have good psychometric properties[34]

For peer review only - http://bmjopen.bmj.com/site/about/guidelines.xhtml

	Summary of Diabetes self-care activities questionnaire SDSCA[35]<br>SDSCA is a diabetes self-care activities questionnaire focusing on general diet, diabetes-specific diet, physical activity, blood-glucose testing, foot care, and smoking.
**Satisfaction with intervention**	Chronic Illness Resources survey (CIRS)[36]<br>CIRS is a questionnaire to represent patient's received support. Individual's support for behavioural-specific disease management is assessed: proximal support e.g. friend and family and distal factor e.g. neighbourhood or community.
	Modified Medical Interview Satisfaction scale (MISS-21)[37]<br>MISS-21 is a questionnaire to measure patient satisfaction with patient and health care professional communication/consultation.

Data collection and follow-up

Each participant will be involved in the study for 12 months after taking consent and baseline data. The trial is expected to start January 2020 and finish October 2021.

Data collection methods will include:

a) Questionnaires: Questionnaire data will be collected face-to-face by research assistants for the full sample at baseline, 6 and 12 months at the community hospital where participants are recruited (Table 1). A custom-designed form linked to RedCap will be used to collect, validate, verify, and store respondents' data where possible or else data will be collected via paper forms and double-entered into the databases. All data files and databases will be password protected.

For peer review only - http://bmjopen.bmj.com/site/about/guidelines.xhtml

b) Biological samples: Blood samples will be collected at baseline, 6 and 12 months, to measure fasting blood glucose, HbA1c and lipids, coordinating where possible with the annual routine tests offered to patients to reduce duplication. All blood samples will be administered from participants by trained phlebotomists. Data will be linked to the participant information using a unique respondent ID, which will be assigned to all study participants.

c) Interviews: During the delivery of the intervention, a process evaluation using a subset of participants will be followed up using in-depth interviews and focus group discussions. These will be audio-recorded. Data will be collected using a range of qualitative methods: a) one-to-one interviews, and focus group discussions with nurses, health volunteers, people with diabetes and their carers (5-10 focus groups, and 20 semi-structured interviews) and b) ethnography through direct observations including video recordings of intervention delivery, and unstructured interviews with clinical managers and policy makers. The data collected will be used to capture the range of experiences of the intervention, and identify unanticipated pathways to generate new theories as well as exploring the scalability of the intervention.

Trial follow-up appointments

The research team will hold weekly briefings with the study coordinators to generate a list of priority areas and loss to follow-up participant lists. Arrangements to follow-up participants who have not turned up for their appointment will be made, with attempts to contact participants through SMS, phone calls, or house visits. Participants will be declared loss to follow-up if they do not show for a month and are untraceable.

For peer review only - http://bmjopen.bmj.com/site/about/guidelines.xhtml

Data management

A data collection protocol will be developed, and the study coordinator in Thailand will provide training to fieldworkers before data collection commences. Validation will be performed a random sample of questionnaire data by crosschecking with clinic records. Any discrepancies will be followed-up and addressed by field workers, re-contacting participants to clarify as necessary. Using Redcap (https://redcap.med.cmu.ac.th), quantitative data will be entered directly via a form with built-in data checks to minimise transcription errors (or where necessary collected on paper and later double entered into the electronic form). Post entry checks will be conducted by exploring the distribution, ranges, and outliers of each variable. All hospital laboratories have their own internal quality assurance protocols and are also linked to a national external quality assurance mechanism. Fieldworkers will be trained in qualitative methods and an interview schedule will be devised.

Statistical analysis

1. Quantitative analysis

Available outcome data will be analysed on an intention-to-treat basis. Potential clustering of outcomes (HbA1c at 12 months and CVD risk score at 12 months) at the level of community primary care units will be accounted for using random intercept models.   and adjusted for baseline values. To improve precisions of the estimates, outcomes will be adjusted for their baseline values. In case of baseline imbalances of relevant covariates (e.g. age, education level, body mass index), judged by statistical significance at $p<0.05$, we will conduct a secondary analysis adjusting for these covariates.

For peer review only - http://bmjopen.bmj.com/site/about/guidelines.xhtml

2. Qualitative analysis

Qualitative data from interviews, focus groups and direct observations be will transcribed and analysed using NVivo software. The data will be analysed using a descriptive, phenomenological approach to understand participants' experiences and interpret them within their respective cultural contexts. Comparative analysis will compare and contrast these themes across participants. Deviant cases will be actively sought throughout the analysis and emerging ideas and themes modified in response. In addition, thematic analysis will be used to inform elements of scalability and to produce a set of considerations in making decisions about the scalability of the intervention.

3. Cost-effective analysis

Data for economic evaluation (resource usage and quality of life using EQ-5D) will be obtained prospectively alongside the trial. We will aim to capture all health service contacts, as well as out-of-pocket expenses and medication use. Educator training costs will be included, as well as minimal intervention material costs (as most will be made available freely after the trial). Utility values from EQ-5D will be derived using a Thai tariff. Incremental cost utility will be estimated from the Thai health system and societal perspectives, to provide incremental cost-effectiveness ratio and probability of being cost-effective at Thai government's willingness to pay threshold of 160,000 baht/QALY.

For peer review only - http://bmjopen.bmj.com/site/about/guidelines.xhtml

For peer review only

ETHICS AND DISSEMINATION

This study is to be conducted according to the international standards of Good Clinical Practice (International Conference on Harmonization guidelines), Declaration of Helsinki, and International Ethical Guidelines for Biomedical Research Involving Human Subjects, applicable national government regulations, and institutional research policies and procedures. All investigators received GCP training at the onset of the study. Ethical approval was obtained prior to commencement of the project from Chiang Mai University [No 326/2018] and the London School of Hygiene & Tropical Medicine [16113/RR/12850].The study protocol, informed consent form, participant's information sheet and other relevant information has been approved. Any future amendments of the protocol shall be submitted to and approved by the Institutional Review Board (IRB) before implementation

Trial monitoring and oversight

The role of the Trial Steering Committee (TSC) is to provide overall supervision of the trial. The TSC will meet every six months. The TSC will include experts in the field of DSME, health psychology and clinical trials, as well as an independent Chair. In addition, we will have patient and carer representatives and policy representation.

Dissemination

Expected output and impact research findings will be disseminated to scientific audiences at major conferences and published in high-impact, open-access scientific journals; planned publications include those on intervention development, primary trial results, and process evaluation, and health systems analysis, at a minimum. This study is expected to have a major

For peer review only - http://bmjopen.bmj.com/site/about/guidelines.xhtml

policy impact due to the close involvement of a key policy maker in the project. Towards the end of the study a dedicated workshop will be held with key governmental stakeholders to disseminate the recommended model for DSME implementation in Thailand and encourage inclusion of a large-scale scientific evaluation into any national implementation of the scheme.

Contributors: CA, SS, OQ, PM, SK were involved in conception and trial design. CA, KP, PM and SK wrote the first draft of the trial design proposal. KR, WT, KH, KK were involved in critical revision of the trial design proposal. All authors contributed to finalising the trial protocol. CA, KP, IPN, CP, AH, NW were involved with preparation of the manuscript for submission. All authors reviewed the manuscript and approved the manuscript for publication.

Funding

This study is supported by UK Medical Research Council (MRC) grant number [MR/R020876/1] and the Thailand Research Fund (TRF) grant number [DBG6180007].

Data availability

Data will be shared upon reasonable request

Patient and Public Involvement

Patients or the public WERE NOT involved in the design, or conduct, or reporting, or dissemination plans of our research

For peer review only - http://bmjopen.bmj.com/site/about/guidelines.xhtml

Trial registration and version

The trial has been registered (ClinicalTrials.gov ID NCT03938233 Version 3 last update January 18,2020)

Trial sponsor

This study is sponsored by London School of Hygiene & Tropical Medicine, Keppel Street, London WC1E 7HT, UK [16113].

Competing interests

None declared

Ethics approval

The study has been approved by the Chiang Mai University Research Ethics Committee [326/2018] and the London School of Hygiene & Tropical Medicine [16113/RR/12850].

Figure 1 Legend: Trial flow diagram

REFERENCES

1. Deerochanawong C, Ferrario A. Diabetes management in Thailand: a literature review of the burden, costs, and outcomes. Glob Health 2013; 9: 11.
2. International Diabetes Federation. IDF Diabetes Atlas Seventh Edition. 2015.
3. Effect of intensive blood-glucose control with metformin on complications in overweight patients with type 2 diabetes (UKPDS 34). The Lancet 1998; 352: 854–65.
4. Gaede P, Vedel P, Larsen N, Jensen GVH, Parving H-H, Pedersen O. Multifactorial intervention and cardiovascular disease in patients with type 2 diabetes. N Engl J Med 2003; 348: 383–93.

For peer review only

For peer review only - http://bmjopen.bmj.com/site/about/guidelines.xhtml

5. Rangsin R. An assessment of quality of care among patients diagnosed with type 2 diabetes and hypertension visiting hospitals of ministry of public health and Bangkok Metropolitan Administration in Thailand. 2012.

6. Auemaneekul N, Chandanasotthi P, Rawiworrakul T, et al. Community partnership, context-based intervention and diabetes control in Thailand. J Public Health 2016; 46: 236–47.

7. Haas L, Maryniuk M, Beck J, et al. National standards for diabetes self-management education and support. Diabetes Educ 2012; 38: 619–629.

8. Chrvala CA, Sherr D, Lipman RD. Diabetes self-management education for adults with type 2 diabetes mellitus: A systematic review of the effect on glycemic control. Patient Educ Couns 2016; 99: 926–43.

9. Pillay J, Armstrong MJ, Butalia S, et al. Behavioral programmes for type 2 diabetes mellitus: A systematic review and network meta-analysis. Ann Intern Med 2015; 163: 848.

10. Doyle G, O'Donnell S, Quigley E, et al. Patient level cost of diabetes self-management education programmes: an international evaluation. BMJ Open 2017; 7: e013805.

11. Fisher EB, Boothroyd RI, Elstad EA, et al. Peer support of complex health behaviors in prevention and disease management with special reference to diabetes: Systematic reviews. Clin Diabetes Endocrinol 2017; 3. DOI:10.1186/s40842-017-0042-3.

12. Dale JR, Williams SM, Bowyer V. What is the effect of peer support on diabetes outcomes in adults? A systematic review. Diabet Med 2012; 29: 1361–77.

For peer review only

For peer review only - http://bmjopen.bmj.com/site/about/guidelines.xhtml

13. Norris SL, Chowdhury FM, Van Le K, et al. Effectiveness of community health workers in the care of persons with diabetes. Diabet Med J Br Diabet Assoc 2006; 23: 544–56.

14. World Health Organization. Peer support programmes in diabetes: report of a WHO consultation 5-7 November 2007. Geneva, Switzerland: WHO, 2008.

15. Fisher EB, Boothroyd RI, Coufal MM, et al. Peer support for self-management of diabetes improved outcomes in international settings. Health Aff (Millwood) 2012; 31: 130–9.

16. Trento M, Gamba S, Gentile L, et al. Rethink Organization to iMprove Education and Outcomes (ROMEO): A multicenter randomized trial of lifestyle intervention by group care to manage type 2 diabetes. Diabetes Care 2010; 33: 745–7.

17. Werfalli M, Raubenheimer P, Engel M, et al. Effectiveness of community-based peer-led diabetes self-management programme (COMP-DSMP) for improving clinical outcomes and quality of life of adults with diabetes in primary care settings in low and middle-income countries (LMIC): A systematic review and meta-analysis. BMJ Open 2015; 5: e007635.

18. Keeratiyutawong P, Hanucharurnkl S, Melkus G, Vorapongsathon OPT. Effectiveness of a self-management programme for Thais with type 2 diabetes. Thai J Nurs Res 2006; 10: 85–97.

19. Craig P, Dieppe P, Macintyre S, Michie S, Nazareth I, Petticrew M. Developing and evaluating complex interventions: the new Medical Research Council guidance. BMJ 2008; a1655.

For peer review only - http://bmjopen.bmj.com/site/about/guidelines.xhtml

20. Wattana C, Srisuphan W, Pothiban L, Upchurch SL. Effects of a diabetes self-management programme on glycaemic control, coronary heart disease risk, and quality of life among Thai patients with type 2 diabetes. Nurs Health Sci 2007; 9: 135–41.

21. Wan EYF, Fung CSC, Wong CKH, Chin WY, Lam CLK. Association of hemogloblin A1c levels with cardiovascular disease and mortality in Chinese patients with diabetes

22. Khunti K, Gray LJ, Skinner T, et al. Effectiveness of a diabetes education and self-management programme (DESMOND) for people with newly diagnosed type 2 diabetes mellitus: Three-year follow-up of a cluster randomised controlled trial in primary care. BMJ 2012; 344: e2333–e2333.

23. Tuong W, Larsen ER, Armstrong AW. Videos to influence: A systematic review of effectiveness of video-based education in modifying health behaviors. J Behav Med 2014; 37: 218–33.

24. Moore GF, Audrey S, Barker M, et al. Process evaluation of complex interventions: Medical Research Council guidance. BMJ 2015; 350: h1258–h1258.

25. Kimman M, et al. Validity of the Thai EQ-5D in an occupational population in Thailand. *Qual Life Res* 2013 Aug; 22(6):1499-506.

26. Clinical Practice Guidelines for Diabetes. Diabetes Association of Thailand, 2017. (https://www.dmthai.org/index.php/knowledge/healthcare-providers/cpg/443-guideline-diabetes-care-2017)

27. Aekplakorn A, Bunnag P, Woodward M. et al. Risk score for predicting incident diabetes in the Thai population. *Diabetes Care* 2006 Aug; 29(8):1872-1877

For peer review only

28. Mahatnirunkul S. Tuntipivatanaskul W, Pumpisanchai W, et al. Comparison of the WHOQOL-100 and the WHOQOL-BREF. *Journal of Mental Health of Thailand* 1998; 5: 4-15.

29. Nilchaikovit T. et al. Development of Thai version of hospital anxiety and depression scale in cancer patients. *J Psychiatr Assoc Thailand* 1996; 41(1):18-30.

30. Wongpakaran N, Wongpakaran T. The Thai version of the PSS-10: An investigation of its psychometric properties. *BioPsychoSocial Medicine* 2010: 4:6

31. Rattanawiwatpong P, et al. Validity and reliability of the Thai version of Short Format International Physical Activity Questionnaire (IPAQ). *J Thai Rehabil* 2006; 16(3):147-160.

32. Leelacharas S, et al. Illness perceptions, lifestyle behaviours, social support and cardiovascular risks in people with Hypertension in Urban and Rural Areas of Thailand. Pacific Rim Int J Nur Res 2015; 19

33. Lamsumang, W. The development of the Thai version of the diabetes management self-efficacy scale (T-DMSES) for older adults with type 2 diabetes. State University of New York at Buffalo, New York; 2009.

34. Sangruangake M, Jirapornkul C, Hurst C. Psychometric properties of diabetes management self-efficacy in Thai type 2 diabetes mellitus patients: A multicenter study. *Int J Endocrinol*. 2017;2017:2503156. doi:10.1155/2017/2503156)

35. Keeratiyatawong, P, Hanucharumkul, S., Melkus, G. D. E., Panpakdee, O., Vorapongsathorn T. Effectiveness of a self-management programme for Thais with type 2 diabetes. Thai J Nurse Res. 2006; 10: 85-97

For peer review only - http://bmjopen.bmj.com/site/about/guidelines.xhtml

36. Manit A, et al. Development of needs and resources for self-management assessment instrument in Thais with type 2 diabetes: Cross-cultural adaptation. *J Med Assoc Thai* 2011; Vol. 94 No. 11.

37. Meakin R and Weinman J. The 'Medical Interview Satisfaction Scale' (MISS-21) adapted for British general practice. *Family Practice* 2002; 19:257–263.

For peer review only

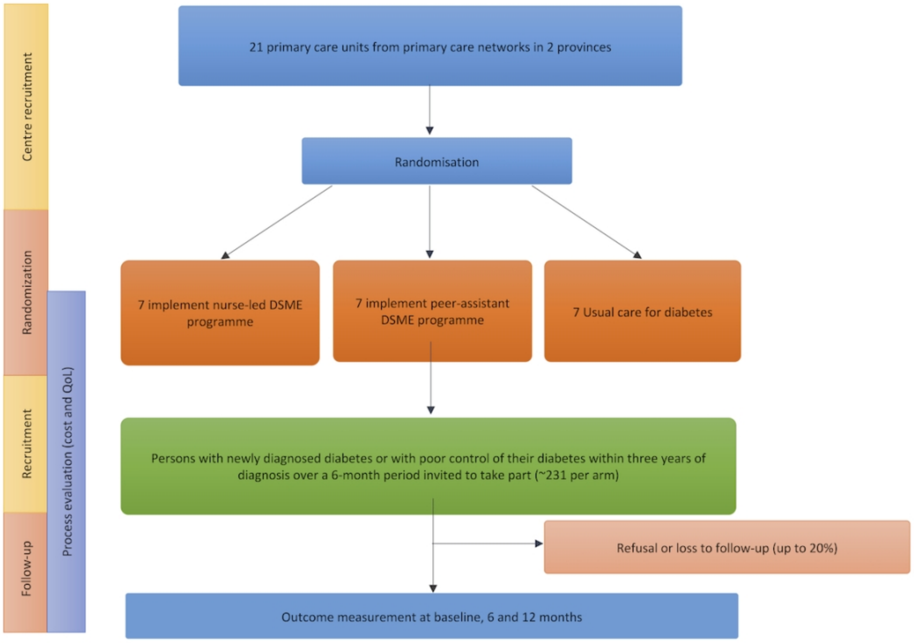

Figure 1: Trial flow diagram

27x20mm (1200 x 1200 DPI)

CHIANG MAI UNIVERSITY

LONDON SCHOOL of HYGIENE &TROPICAL MEDICINE

Participant Information Sheet

Title of Project: Delivering Diabetes Self-Management Education in Thailand

Introduction

We would like to invite you to take part in a research study. Joining the study is entirely up to you. Before you decide, you need to understand why the research is being done and what it would involve. One of our team will go through this information sheet with you, and answer any questions you may have. Ask questions if anything you read is not clear or you would like more information. Please feel free to talk to others about the study if you wish. Take time to decide whether or not to take part.

What is the purpose of the study?

The London School of Hygiene and Tropical Medicine (LSHTM) are conducting research for people with type 2 diabetes in Chiang Mai Thailand who have recently been diagnosed. The purpose of this study is to provide diabetes self-management education (DSME) by trained nurses and peer health care volunteers for those recently diagnosed.

This is a 3 arm cluster randomised trial where your service provider has been randomised to one of the following 3 interventions: 1) usual treatment 2) Nurse led DSME 3) Peer led DSME.

Why have I been asked to take part?

You have been invited because your healthcare provider is involved in the study and has identified you as being newly diagnosed with type 2 diabetes.

Do I have to take part?

No. It is up to you to decide to take part or now. If you don't want to take part, that's ok. Your doctor will still care for you and your decision will not affect the quality of care you receive.

We will discuss the study together and give you a copy of this information sheet. If you agree to take part, we will then ask you to sign a consent form.

What will happen to me if I take part?

Enrolling you in the study

If you are interested in taking part the research assistant will go through the information sheet with you. If you are happy with the information provided you may complete the consent form.

At this point you will also have the opportunity to raise with the researcher any questions you might have about the study. You do not have to enter the study unless you feel completely happy with what you are being asked to do.

For peer review only - http://bmjopen.bmj.com/site/about/guidelines.xhtml

For peer review only

CHIANG MAI UNIVERSITY

LONDON SCHOOL of HYGIENE &TROPICAL MEDICINE

Participating in a diabetes education programme

You will be invited to attend a diabetes self-management educational programme. This programme will consist of a number of sessions delivered as monthly meetings led by community health volunteers or nurses. Short films about living with diabetes will be shown to introduce key topics.

The education and skills training will be followed by an open discussion session to discuss common challenges and solutions, set lifestyle goals and seek advice.

Collecting information

If you are happy to proceed with the study the researcher will then ask a series of questions relating to your health. This will be through completing a questionnaire booklet. These baseline questions will take about twenty minutes to complete. We will also take blood samples as the beginning of the study from trained phlebotomists.

Each participant will be compensated 200 baht for each of the baseline, 6 month and 12 month visit.

Follow up

Six months and twelve months after you have entered the study we will contact you again to ask you a further set of questions and blood samples to see how you are feeling now.

How is taking part in the study different from usual care?

Additional support and education may be provided through the study through the self-management education programme.

Whilst you are taking part in the study you will continue to be looked after by your healthcare providers, as normal. No treatment will be withheld from you during the course of this study.

The researcher will want you to complete some questionnaires at the beginning of the study and after six and twelve months. You may be asked to take part in an interview with a researcher.

What are the possible risks and disadvantages?

It will take some time to complete the questionnaire, around twenty minutes to complete. If you agree to be further interviewed, then this will also mean time will be needed to attend the interview. You may find minor discomfort from the taking blood samples. We will have trained phlebotomists to take the blood sample.

What are the possible benefits?

We cannot promise the study will help you but the information we get from the study will help our knowledge and understanding of this research area within diabetes self-management education in Thailand.

For peer review only - http://bmjopen.bmj.com/site/about/guidelines.xhtml

CHIANG MAI UNIVERSITY

LONDON SCHOOL of HYGIENE &TROPICAL MEDICINE

What if something goes wrong?
If you have a concern about any aspect of this study, you should ask to speak to the researchers who will do their best to answer your questions <+66(0)616852307>. If you remain unhappy and wish to complain formally, you can do this by contacting <if LSHTM is the sponsor: Patricia Henley at rgio@lshtm.ac.uk or +44 (0) 20 7927 2626>

The London School of Hygiene and Tropical Medicine holds insurance policies which apply to this study. If you experience harm or injury as a result of taking part in this study, you may be eligible to claim compensation.

Can I change my mind about taking part?
Yes. You can withdraw from the study at any time. You just need to tell your doctor that you don't want to be in the study anymore. Your doctor will still care for you.

If you withdraw from the study we will destroy all your identifiable samples/ tape recorded interviews, but we will need to use the data collected on you up to your withdrawal

Or

You can withdraw from treatment but keep in contact with us to let us know your progress. Information collected may still be used. Any stored blood or tissue samples that can still be identified as yours will be destroyed if you wish, or will continue to be stored for further research.

What will happen to information collected about me?
All information collected about you will be kept private. Only the study staff and authorities who check that the study is being carried out properly will be allowed to look at information about you. Data may be sent to other study staff in London or in Chiang Mai but this will be anonymised. This means that any information about you which leaves the hospital, will have your name and address removed so that you cannot be recognised.

Your doctor will send some details about you to the study team in Chiang Mai University who will store it securely. Your personal details will be kept in a different safe place to the other study information and will be destroyed within 10 years of the end of the study.

At the end of the project, the study data will be archived at Chiang Mai University. The data will be made available to other researchers worldwide for research and to improve medical knowledge and patient care. Your personal information will not be included and there is no way that you can be identified.

What will happen to the results of this study?
The study results will be published in a medical journal so that other doctors can learn from them. Your personal information will not be included in the study report and there is no way that you can be identified from it.

Who is organising and funding this study?
London School of Hygiene & Tropical Medicine is the sponsor for the research and they have full responsibility for the project including the collection, storage and analysis of your data.
This study is funded by the Medical Research Council UK and The Thailand Research Fund

Who has checked this study?
All research involving human participants is looked at by an independent group of people, called a Research Ethics Committee, to protect your interests. This study has been reviewed and given favourable opinion by The London

For peer review only - http://bmjopen.bmj.com/site/about/guidelines.xhtml

School of Hygiene and Tropical Medicine Research Ethics Committee (<ref: 16113>). The Research Ethics Committee, Faculty of Medicine, Chiang Mai University (No 326/2018) has also reviewed the study and have agreed that it is okay for us to ask people to take part.

Further information and contact details

Thank you for taking time to read this information leaflet. If you think you will take part in the study please read and sign the consent form.

If you would like any further information, please contact Dr. Chaisiri Angkurawaranon, MD, who can answer any questions you may have about the study.

Contact details: Chaisiri Angkurawaranon, Department of Family Medicine, Faculty of Medicine, Chiang Mai University. Tel +66(0)616852307, Email: chaisiri.a@cmu.ac.th

What happens if new information becomes available during the study?

Sometimes during a study, new information becomes available about the treatment being studied. If this happens, the research team will tell you and discuss whether you want to continue in the study. If you decide to stop taking part in the study your usual care will continue. If you decide to continue in the study you may be asked to sign an updated consent form. If we think you should withdraw from the study, we will explain the reasons and arrange for your care to continue.

What happens when the study stops?

Very occasionally a study is stopped early. If this happens, the reasons will be explained to you.

Tissue studies:

What will happen to the samples I give?

Blood samples will be used to measure HbA1c and lipids, coordinating where possible with the annual routine tests offered to patients to reduce duplication. Blood will be drawn by a trained phlebotomist when participants come for interview and sent to laboratory for analysis. Data will be linked to the participant information using a unique respondent ID, which will be assigned to all study participants.

Blood samples will be sent to a laboratory within the Chiang Mai area. The results of the blood tests will be provided to your health care professional who will discuss with you if there are any issues.

We may use some of the samples collected for future studies. These will be anonymised when stored, and all future research using these samples will be reviewed by an independent ethics committee.

For peer review only

For peer review only - http://bmjopen.bmj.com/site/about/guidelines.xhtml

LONDON SCHOOL of HYGIENE &TROPICAL MEDICINE

CONSENT FORM FOR PARTICIPANT AND REPRESENTATIVE

Title of Project: A scalable solution for delivery of Diabetes Self-Management Education in Thailand

Name of PI/Researcher responsible for project: Chaisiri Angkurawaranon

**Statement**	**Please initial or thumbprint* each box**
I confirm that I have read and understood the information sheet dated 08.10.2018 (version 1.0) for the above named study. I have had the opportunity to consider the information, ask questions and have these answered satisfactorily.	
I understand that my consent is voluntary and that I am free to withdraw this consent at any time without giving any reason and without my medical care or legal rights being affected.	
I understand that relevant sections of my/the participant's medical notes and data collected during the study may be looked at by authorised individuals from Chiang Mai University, where it is relevant to my taking part in this research. I give permission for these individuals to have access to these records.	
I understand that data about/from me/the participant may be shared via a public data repository or by sharing directly with other researchers, and that I will not be identifiable from this information	
I understand that the tissue sample collected from me will be used to support other research in the future, and may be shared anonymously with other researchers, for their ethically-approved projects	
I give permission for a copy of this consent form, which contains my personal information, to be made available to the Trial Coordinating Centre for monitoring purposes only.	
I agree to my health care provider being informed of my participation in the study.	
I agree to me taking part in the above named study.	

Printed name of participant/Representative	Signature of participant/Representative (or thumbprint/mark if unable to sign)	Date

Printed name of person obtaining consent	Signature of person obtaining consent	Date

The participant is unable to sign. As a witness, I confirm that all the information about the trial was given and the participant/representative consented to taking part *(*only required if the participant/representative is unable to read or write)*

Printed name of impartial witness*	Signature of impartial witness*	Date

A copy of this informed consent document has been provided to the participant.

Centre Number:
Study Number:
Participant Identification Number:

For peer review only

For peer review only - http://bmjopen.bmj.com/site/about/guidelines.xhtml

แบบขอรับความยินยอมเข้าร่วมโครงการ (Consent ข้อมูลสำหรับผู้ป่วย)

ชื่อโครงการศึกษาวิจัย : "การพัฒนาทางออกเพื่อการขยายการบริการที่เพิ่มความสามารถในการดูแลตนเองของผู้ที่เป็นเบาหวานในประเทศไทย"

หมายเลขโครงการศึกษาวิจัย : FAM-2561-05594

ผู้ให้ทุนสนับสนุนการวิจัย : สำนักงานคณะกรรมการส่งเสริมวิทยาศาสตร์ วิจัยและนวัตกรรม (สกสว.)

แพทย์ผู้วิจัยหลัก : อ.ดร.นพ.ชัยสิริ อังกุระวรานนท์

ท่านได้รับการเชื้อเชิญให้เข้าร่วมการศึกษาวิจัยนี้เนื่องจากท่านได้รับการวินิจฉัยว่าเป็นโรคเบาหวานที่เพิ่งได้รับการวินิจฉัยมาไม่เกิน 1 ปี หรือเป็นผู้ป่วยเบาหวานที่ยังไม่สามารถควบคุมน้ำตาลได้ ขอให้ท่านกรุณาอ่านข้อมูลข้างล่างก่อน (หรือผู้วิจัยได้อ่านให้ท่านรับทราบ) หากท่านมีข้อข้องใจใดๆ เกี่ยวกับการศึกษาวิจัยนี้ และสิทธิของท่าน กรุณาซักถามจากแพทย์ผู้ทำการศึกษาวิจัย หรือ ผู้ช่วยแพทย์ที่ทำการศึกษาวิจัย ซึ่งจะเป็นผู้สามารถให้ความกระจ่างแก่ท่านได้ หากท่านตัดสินใจเข้าร่วมการศึกษาวิจัย ท่านจะได้รับเอกสารข้อมูลสำหรับผู้เข้าร่วมโครงการวิจัยและสำเนาใบยินยอมที่ท่านเซ็นชื่อกำกับเก็บไว้ 1 ฉบับ

การศึกษาวิจัยนี้เกี่ยวกับเรื่องอะไร

เนื่องจากในปัจจุบันนี้มีผู้ป่วยเป็นโรคเบาหวานจำนวนมากขึ้น และพบว่าการป่วยเป็นโรคเบาหวานนี้สัมพันธ์กับภาวะแทรกซ้อนทางสุขภาพมากมาย เพื่อให้เป็นประโยชน์ต่อการพัฒนาการดูแลรักษาผู้ป่วยเบาหวานในอนาคต ผู้วิจัยจึงสนใจที่จะศึกษาว่าการเป็นโรคเบาหวานนั้นสัมพันธ์กับการเปลี่ยนแปลงระดับชีวภาพในร่างกายอย่างไร โดยผ่านการตรวจเลือดและอุจจาระ และเพื่อศึกษาว่าการจัดทำโครงการให้ความรู้ในการดูแลตนเองของผู้ป่วยเบาหวานโดยใช้สื่อมัลติมีเดียเข้าช่วยเหลือมีผลลัพธ์ของการรักษาที่ดีขึ้นได้หรือไม่ การศึกษานี้จะรวบรวมผู้ป่วยประมาณ 600 ราย จากโรงพยาบาลชุมชนที่ให้การรักษาแบบปฐมภูมิ ในจังหวัดเชียงใหม่ และ ลำปาง

ท่านจะต้องปฏิบัติตัวอย่างไร

หากท่านตัดสินใจเข้าร่วมการศึกษาวิจัยนี้ท่านจะถูกขอร้องให้เซ็นชื่อลงในใบยินยอมท่านจะได้รับการซักประวัติเกี่ยวกับโรคประจำตัวและการรักษาที่ได้รับ ได้ตรวจร่างกาย และการเจาะเลือด

เพื่อเป็นข้อมูลเริ่มต้นก่อนการรักษา โดยทั้งนี้ท่านจะได้รับการดูแลตามปกติควบคู่ไป

จากนั้นท่านจะได้รับการอบรมเพื่อพัฒนาศักยภาพในการดูแลตนเองในโรคเบาหวานของท่านโดยทีมดูแลสุขภาพ ซึ่งหน่วยบริการของท่านจะถูกสุ่มให้อยู่ 1 ใน 3 กลุ่ม อันได้แก่ กลุ่มทดลอง1 กลุ่มทดลอง2 และกลุ่มควบคุม โดยทั้ง 3 กลุ่มจะได้รับข้อมูลและการดูแลพื้นฐานเช่นเดียวกัน แต่แตกต่างกันในรูปแบบของการให้การอบรมในการดูแลตนเองของผู้ป่วยเบาหวาน ซึ่งจะใช้เวลานาน ไม่เกิน ครึ่งวัน โดย

For peer review only - http://bmjopen.bmj.com/site/about/guidelines.xhtml

- กลุ่มที่1 อบรมโดยบุคลากรทางการแพทย์ พยาบาล หรือ เจ้าหน้าที่สาธารณสุข
- กลุ่มที่2 อบรมโดยกลุ่มเพื่อนช่วยเพื่อนหรืออสม.ที่ผ่านการฝึกอบรมด้านการเพิ่มศักยภาพในการดูแลตนเองในโรคเบาหวาน
- กลุ่มที่ 3 ได้รับความรู้จากการดูแลตามมาตรฐานของกระทรวงสาธารณสุข

ซึ่งทั้งสามกลุ่ม จะมีการนัด หรือ ติดตาม ความก้าวหน้าพฤติกรรมการดูแลตนเอง ทุกๆ 1-3 เดือน

การติดตามผลเลือดและตอบแบบสอบถาม จะถูกทำในครั้งแรกข้างต้น และอีก 2 ครั้ง คือ ที่ 6 เดือนและที่ 12 เดือนนับจากครั้งแรกที่อบรม โดยทั้ง 3 ครั้ง ท่านจะถูกเจาะเลือดเพื่อส่งตรวจปริมาตรทั้งสิ้นครั้งละ 40 มิลลิลิตร

ความเสี่ยงจากการเข้าร่วมการวิจัยนี้

ความเสี่ยงจากการเจาะเลือด - ท่านอาจรู้สึกหน้ามืด เป็นลม ปวดบริเวณที่เจาะ หรือมีจ้ำเลือดบริเวณที่เจาะ มีความเสี่ยงน้อยมากที่จะเกิดการติดเชื้อจากการเจาะเลือดเพราะเราใช้เข็มเจาะเลือดที่ปราศจากเชื้อและใช้ครั้งเดียวทิ้ง การเจาะเลือดทั้งหมดอยู่ในสถานพยาบาล และหากมีปัญหาจะได้รับการดูแลทันที

ท่านจะได้ประโยชน์อะไรจากการศึกษานี้

ท่านจะได้รับความรู้เพื่อนำไปใช้ในการดูแลตนเองเพื่อควบคุมโรคเบาหวานของท่าน และผลสรุปที่ได้จากการศึกษานี้จะเป็นประโยชน์ต่อผู้ป่วยรายอื่นในอนาคต

ค่าใช้จ่ายในการเข้าร่วมวิจัย

ท่านจะไม่ต้องเสียค่าใช้จ่ายเพิ่มเติมเกี่ยวกับการวิจัยนอกเหนือจากค่ารักษาปกติที่ควรจะเป็นซึ่งท่านสามารถใช้สิทธิในการเบิกจ่ายได้ตามสิทธิการรักษาปกติของท่าน

ค่าตอบแทน

สำหรับการมาตรวจติดตามที่เพิ่มจากการตรวจรักษาตามปกติ จำนวน 5 ครั้ง ซึ่งจะมีการตรวจเลือด รวม 3 ครั้ง ท่านจะได้รับค่าเดินทางและค่าเสียเวลาครั้งละ 100 บาท (รวม 500 บาทต่อคน ตลอดระยะเวลาโครงการ 1 ปี)

For peer review only - http://bmjopen.bmj.com/site/about/guidelines.xhtml

หากท่านได้รับบาดเจ็บจากการเข้าร่วมการศึกษาวิจัย

หากท่านได้ปฏิบัติตามคำแนะนำของแพทย์ผู้วิจัยแล้ว กระบวนการต่าง ๆ ในการวิจัยทำให้ท่านได้รับบาดเจ็บ เมื่อผู้วิจัยได้รับแจ้งจากท่าน ท่านจะได้รับการส่งต่อเพื่อรักษาภาวะดังกล่าวทันที

ท่านจะทำอย่างไรหากท่านไม่ต้องการเข้าร่วมการศึกษาวิจัย หรือเปลี่ยนใจระหว่างร่วมศึกษาวิจัย

ท่านไม่จำเป็นต้องเข้าร่วมการศึกษาวิจัยนี้หากท่านไม่สมัครใจ หลังจากท่านตัดสินใจจะเข้าร่วมการศึกษาแล้ว ท่านสามารถจะถอนตัวได้ตลอดเวลา การตัดสินใจของท่านจะไม่มีผลต่อการรักษาในอนาคต หรือการดูแลอื่นใดหากท่านไม่ต้องการเข้าร่วมการศึกษาหรือต้องการหยุดการศึกษา ณ เวลาใดก็ตาม แพทย์ของท่านจะอธิบายให้ทราบถึงการรักษาอื่น ๆ ซึ่งเป็นทางเลือกที่มีอยู่ขณะนี้ ผู้วิจัยอาจจะตัดสินใจยกเลิกท่านจากการศึกษา หากเห็นว่าจะเป็นประโยชน์สำหรับท่านมากกว่า

ใครจะรู้บ้างว่าท่านเข้าร่วมการศึกษานี้

แพทย์ประจำตัวท่าน (แพทย์เวชปฏิบัติทั่วไป) ควรจะได้รับทราบว่าท่านตัดสินใจเข้าร่วมการศึกษาวิจัยนี้ ข้อมูลของท่านที่ถูกบันทึกไว้ระหว่างการศึกษา เช่นเดียวกับข้อมูลที่เกี่ยวข้องจากแฟ้มเวชระเบียนของโรงพยาบาล คลินิก บริษัทฯ หรือข้อมูลอื่น ๆ จะถูกเก็บไว้เป็นความลับตลอดเวลา คณะกรรมการจริยธรรมการวิจัยสามารถที่จะขอตรวจสอบข้อมูลเหล่านี้ได้ โดยข้อมูลเหล่านี้จะยังเก็บรักษาไว้เป็นเรื่องลับเฉพาะ

การปกป้องรักษาข้อมูล : ข้อมูลใดบ้างที่จะถูกเก็บรวบรวมไว้จากการศึกษานี้

ข้อมูลที่ถูกเก็บรวบรวมนั้นจะมีเฉพาะในส่วนที่เกี่ยวข้องกับการศึกษาเพื่อวัตถุประสงค์ทางการวิจัยทางการแพทย์ โดยจะไม่มีการอ้างถึงข้อมูลส่วนตัวของท่าน อันได้แก่ชื่อท่าน ในรายงานหรือวารสารใด ๆ หากท่านตกลงใจเข้าร่วมการศึกษา ท่านยินยอมที่จะไม่จำกัดการให้ข้อมูลที่เป็นส่วนตัวยกเว้นในกรณีที่ขัดต่อสิทธิส่วนบุคคลภายใต้กฎหมายคุ้มครองลิขสิทธิ์ส่วนบุคคล

หากท่านมีคำถามเกี่ยวกับการศึกษานี้ท่านสามารถติดต่อใครได้บ้าง

หากท่านมีคำถามหรือมีความวิตกกังวลเกี่ยวกับการศึกษาวิจัยนี้ หรือสงสัยว่าท่านกำลังได้รับบาดเจ็บจากการเข้าร่วมการวิจัยนี้ กรุณาติดต่อ คุณกุลญาภา อยู่นัด ได้ที่ ภาควิชาเวชศาสตร์ครอบครัว โทรศัพท์ที่ทำงาน 053-936362 (ในเวลาราชการ)

ส่วนแสดงความยินยอม

โดยการลงนามในหนังสือยินยอมฉบับนี้ ท่านยอมรับว่าได้อ่านเอกสารฉบับนี้แล้วและได้รับคำอธิบายเกี่ยวกับการศึกษาวิจัยนี้ รวมถึงได้รับคำตอบเกี่ยวกับข้อสงสัยต่าง ๆ ที่ท่านมีจากผู้วิจัยแล้ว

For peer review only - http://bmjopen.bmj.com/site/about/guidelines.xhtml

ท่านตกลงใจที่จะเข้าร่วมในการศึกษาวิจัยนี้ และมีสิทธิ์ที่จะถอนตัวจากการศึกษาวิจัยนี้ได้ทุกเมื่อ โดยไม่มีผลต่อการรักษาในอนาคต โดยการลงนามนี้ท่านไม่ได้สละสิทธิ์ใด ๆ ที่พึงมีทางกฎหมาย

ทั้งนี้ ท่านยินยอมที่จะเข้ารับการอบรมในโครงการวิจัย ตอบแบบสอบถามและ

ลายมือชื่ออาสาสมัคร ______________________________ วัน-เดือน-ปี_______________

(________________________________)

ลายมือชื่อเจ้าหน้าที่ผู้ให้ข้อมูล ______________________________ วัน-เดือน-ปี_______________
ณ สถานพยาบาล

(________________________________)

พยาน ______________________________ วัน-เดือน-ปี_______________

(________________________________)

Consent form WP3 intervention phase DSME 5 June 2018

For peer review only

SPIRIT

STANDARD PROTOCOL ITEMS: RECOMMENDATIONS FOR INTERVENTIONAL TRIALS

SPIRIT 2013 Checklist: Recommended items to address in a clinical trial protocol and related documents*

Section/item	ItemNo	Description	Page/Line number
**Administrative information**			
Title	1	Descriptive title identifying the study design, population, interventions, and, if applicable, trial acronym	1
Trial registration	2a	Trial identifier and registry name. If not yet registered, name of intended registry	Line 357
	2b	All items from the World Health Organization Trial Registration Data Set	-
Protocol version	3	Date and version identifier	Lines 357-358
Funding	4	Sources and types of financial, material, and other support	Lines 344-346
Roles and responsibilities	5a	Names, affiliations, and roles of protocol contributors	Page 1 and Lines 338-342
	5b	Name and contact information for the trial sponsor	Lines 359-360
	5c	Role of study sponsor and funders, if any, in study design; collection, management, analysis, and interpretation of data; writing of the report; and the decision to submit the report for publication, including whether they will have ultimate authority over any of these activities	Lines 344-353

For peer review only

	5d	Composition, roles, and responsibilities of the coordinating centre, steering committee, endpoint adjudication committee, data management team, and other individuals or groups overseeing the trial, if applicable (see Item 21a for data monitoring committee)	Lines 322-326
**Introduction**			
Background and rationale	6a	Description of research question and justification for undertaking the trial, including summary of relevant studies (published and unpublished) examining benefits and harms for each intervention	Page 4 and 5
	6b	Explanation for choice of comparators	Lines 99-103
Objectives	7	Specific objectives or hypotheses	Lines 92-94, 217-226
Trial design	8	Description of trial design including type of trial (eg, parallel group, crossover, factorial, single group), allocation ratio, and framework (eg, superiority, equivalence, noninferiority, exploratory)	Lines 98-103
**Methods: Participants, interventions, and outcomes**			
Study setting	9	Description of study settings (eg, community clinic, academic hospital) and list of countries where data will be collected. Reference to where list of study sites can be obtained	Lines 105-117
Eligibility criteria	10	Inclusion and exclusion criteria for participants. If applicable, eligibility criteria for study centres and individuals who will perform the interventions (eg, surgeons, psychotherapists)	Lines 119-129

For peer review only - http://bmjopen.bmj.com/site/about/guidelines.xhtml

Interventions	11a	Interventions for each group with sufficient detail to allow replication, including how and when they will be administered	Lines 157-187
	11b	Criteria for discontinuing or modifying allocated interventions for a given trial participant (eg, drug dose change in response to harms, participant request, or improving/worsening disease)	n/a
	11c	Strategies to improve adherence to intervention protocols, and any procedures for monitoring adherence (eg, drug tablet return, laboratory tests)	Lines 192-197
	11d	Relevant concomitant care and interventions that are permitted or prohibited during the trial	n/a
Outcomes	12	Primary, secondary, and other outcomes, including the specific measurement variable (eg, systolic blood pressure), analysis metric (eg, change from baseline, final value, time to event), method of aggregation (eg, median, proportion), and time point for each outcome. Explanation of the clinical relevance of chosen efficacy and harm outcomes is strongly recommended	Lines 217-229
Participant timeline	13	Time schedule of enrolment, interventions (including any run-ins and washouts), assessments, and visits for participants. A schematic diagram is highly recommended (see Figure)	Page 9-11

For peer review only - http://bmjopen.bmj.com/site/about/guidelines.xhtml

Sample size	14	Estimated number of participants needed to achieve study objectives and how it was determined, including clinical and statistical assumptions supporting any sample size calculations	Lines 140-148
Recruitment	15	Strategies for achieving adequate participant enrolment to reach target sample size	n/a
**Methods: Assignment of interventions (for controlled trials)**			
Allocation:			
Sequence generation	16a	Method of generating the allocation sequence (eg, computer-generated random numbers), and list of any factors for stratification. To reduce predictability of a random sequence, details of any planned restriction (eg, blocking) should be provided in a separate document that is unavailable to those who enrol participants or assign interventions	Lines 131-138
Allocation concealment mechanism	16b	Mechanism of implementing the allocation sequence (eg, central telephone; sequentially numbered, opaque, sealed envelopes), describing any steps to conceal the sequence until interventions are assigned	n/a
Implementation	16c	Who will generate the allocation sequence, who will enrol participants, and who will assign participants to interventions	n/a
Blinding (masking)	17a	Who will be blinded after assignment to interventions (eg, trial participants, care providers, outcome assessors, data analysts), and how	n/a

	17b	If blinded, circumstances under which unblinding is permissible, and procedure for revealing a participant's allocated intervention during the trial	
**Methods: Data collection, management, and analysis**			
Data collection methods	18a	Plans for assessment and collection of outcome, baseline, and other trial data, including any related processes to promote data quality (eg, duplicate measurements, training of assessors) and a description of study instruments (eg, questionnaires, laboratory tests) along with their reliability and validity, if known. Reference to where data collection forms can be found, if not in the protocol	Lines 228 Table 2
	18b	Plans to promote participant retention and complete follow-up, including list of any outcome data to be collected for participants who discontinue or deviate from intervention protocols	n/a
Data management	19	Plans for data entry, coding, security, and storage, including any related processes to promote data quality (eg, double data entry; range checks for data values). Reference to where details of data management procedures can be found, if not in the protocol	Lines 264
Statistical methods	20a	Statistical methods for analysing primary and secondary outcomes. Reference to where other details of the statistical analysis plan can be found, if not in the protocol	Lines 277-285
	20b	Methods for any additional analyses (eg, subgroup and adjusted analyses)	Lines 277-285

	20c	Definition of analysis population relating to protocol non-adherence (eg, as randomised analysis), and any statistical methods to handle missing data (eg, multiple imputation)	n/a
**Methods: Monitoring**			
Data monitoring	21a	Composition of data monitoring committee (DMC); summary of its role and reporting structure; statement of whether it is independent from the sponsor and competing interests; and reference to where further details about its charter can be found, if not in the protocol. Alternatively, an explanation of why a DMC is not needed	Lines 322
	21b	Description of any interim analyses and stopping guidelines, including who will have access to these interim results and make the final decision to terminate the trial	n/a
Harms	22	Plans for collecting, assessing, reporting, and managing solicited and spontaneously reported adverse events and other unintended effects of trial interventions or trial conduct	n/a
Auditing	23	Frequency and procedures for auditing trial conduct, if any, and whether the process will be independent from investigators and the sponsor	n/a
**Ethics and dissemination**			
Research ethics approval	24	Plans for seeking research ethics committee/institutional review board (REC/IRB) approval	Lines 364

For peer review only - http://bmjopen.bmj.com/site/about/guidelines.xhtml

Protocol amendments	25	Plans for communicating important protocol modifications (eg, changes to eligibility criteria, outcomes, analyses) to relevant parties (eg, investigators, REC/IRBs, trial participants, trial registries, journals, regulators)	Lines 319-320
Consent or assent	26a	Who will obtain informed consent or assent from potential trial participants or authorised surrogates, and how (see Item 32)	Lines 150-155
	26b	Additional consent provisions for collection and use of participant data and biological specimens in ancillary studies, if applicable	n/a
Confidentiality	27	How personal information about potential and enrolled participants will be collected, shared, and maintained in order to protect confidentiality before, during, and after the trial	Lines 150-155
Declaration of interests	28	Financial and other competing interests for principal investigators for the overall trial and each study site	Lines 362-363
Access to data	29	Statement of who will have access to the final trial dataset, and disclosure of contractual agreements that limit such access for investigators	Lines 348-349
Ancillary and post-trial care	30	Provisions, if any, for ancillary and post-trial care, and for compensation to those who suffer harm from trial participation	n/a

For peer review only - http://bmjopen.bmj.com/site/about/guidelines.xhtml

Dissemination policy	31a	Plans for investigators and sponsor to communicate trial results to participants, healthcare professionals, the public, and other relevant groups (eg, via publication, reporting in results databases, or other data sharing arrangements), including any publication restrictions	Lines 328-336
	31b	Authorship eligibility guidelines and any intended use of professional writers	n/a
	31c	Plans, if any, for granting public access to the full protocol, participant-level dataset, and statistical code	n/a
**Appendices**			
Informed consent materials	32	Model consent form and other related documentation given to participants and authorised surrogates	Attached
Biological specimens	33	Plans for collection, laboratory evaluation, and storage of biological specimens for genetic or molecular analysis in the current trial and for future use in ancillary studies, if applicable	n/a

*It is strongly recommended that this checklist be read in conjunction with the SPIRIT 2013 Explanation & Elaboration for important clarification on the items. Amendments to the protocol should be tracked and dated. The SPIRIT checklist is copyrighted by the SPIRIT Group under the Creative Commons "Attribution-NonCommercial-NoDerivs 3.0 Unported" license.

For peer review only - http://bmjopen.bmj.com/site/about/guidelines.xhtml

BMJ Open

A Scalable Solution for Delivery of Diabetes Self-Management Education in Thailand (DSME-T): A Cluster Randomized Trial Study Protocol

Journal:	*BMJ Open*
Manuscript ID	bmjopen-2020-036963.R2
Article Type:	Protocol
Date Submitted by the Author:	30-Aug-2020

Complete List of Authors:	Angkurawaranon, Chaisiri; Chiang Mai University, Department of Family Medicine, Faculty of Medicine<br>Papachristou Nadal, Iliatha; London School of Hygiene and Tropical Medicine, Faculty of Epidemiology and Population Health<br>Mallinson, Poppy; London School of Hygiene and Tropical Medicine Faculty of Epidemiology and Population Health, Department of Non-communicable Disease Epidemiology<br>Pinyopornpanish, Kanokporn; Chiang Mai University, Department of Family Medicine, Faculty of Medicine<br>Quansri, Orawan; Mahidol University<br>Rerkasem, Kittipan ; Chiang Mai University, Department of Surgery, Faculty of Medicine, Chiang Mai University; Chiang Mai University, Research Institute for Health Sciences<br>Srivanichakorn, Supattra; Royal Thai Government Ministry of Public Health<br>Techakehakij, Win; Lampang Hospital<br>Wichit, Nutchanath; Surat Thani Rajabhat University<br>Pateekhum, Chanapat; Chiang Mai University<br>Hashmi, Ahmar; Chiang Mai University, Department of Family Medicine<br>Hanson, Kara; London School of Hygiene and Tropical Medicine<br>Khunti, Kamlesh; University of Leicester, Department of Health Sciences<br>Kinra, Sanjay; London School of Hygiene and Tropical Medicine

**Primary Subject Heading**:	Diabetes and endocrinology
Secondary Subject Heading:	Evidence based practice, Diabetes and endocrinology, General practice / Family practice
Keywords:	DIABETES & ENDOCRINOLOGY, EDUCATION & TRAINING (see Medical Education & Training), Protocols & guidelines < HEALTH SERVICES ADMINISTRATION & MANAGEMENT

SCHOLARONE™
Manuscripts

For peer review only - http://bmjopen.bmj.com/site/about/guidelines.xhtml

I, the Submitting Author has the right to grant and does grant on behalf of all authors of the Work (as defined in the below author licence), an exclusive licence and/or a non-exclusive licence for contributions from authors who are: i) UK Crown employees; ii) where BMJ has agreed a CC-BY licence shall apply, and/or iii) in accordance with the terms applicable for US Federal Government officers or employees acting as part of their official duties; on a worldwide, perpetual, irrevocable, royalty-free basis to BMJ Publishing Group Ltd ("BMJ") its licensees and where the relevant Journal is co-owned by BMJ to the co-owners of the Journal, to publish the Work in this journal and any other BMJ products and to exploit all rights, as set out in our licence.

The Submitting Author accepts and understands that any supply made under these terms is made by BMJ to the Submitting Author unless you are acting as an employee on behalf of your employer or a postgraduate student of an affiliated institution which is paying any applicable article publishing charge ("APC") for Open Access articles. Where the Submitting Author wishes to make the Work available on an Open Access basis (and intends to pay the relevant APC), the terms of reuse of such Open Access shall be governed by a Creative Commons licence – details of these licences and which Creative Commons licence will apply to this Work are set out in our licence referred to above.

Other than as permitted in any relevant BMJ Author's Self Archiving Policies, I confirm this Work has not been accepted for publication elsewhere, is not being considered for publication elsewhere and does not duplicate material already published. I confirm all authors consent to publication of this Work and authorise the granting of this licence.

For peer review only

A Scalable Solution for Delivery of Diabetes Self-Management Education in Thailand (DSME-T): A Cluster Randomized Trial Study Protocol

Chaisiri Angkurawaranon[1*], Iliatha Papachristou Nadal[2,3], Poppy Alice Carson Mallinson[2,] Kanokporn Pinyopornpanish[1], Orawan Quansri[4], Kittipan Rerkasem[5,6], Supattra Srivanichakorn[7], Win Techakehakij[8], Nutchanath Wichit[9], Chanapat Pateekhum[1], Ahmar Hashmi[1], Kara Hanson[10], Kamlesh Khunti[11], Sanjay Kinra[2]

Author affiliations

[1] Department of Family Medicine, Faculty of Medicine, Chiang Mai University

[2] Faculty of Epidemiology and Population Health, London School of Hygiene and Tropical Medicine

[3] Psychological Medicines, King's College London

[4] ASEAN Institute for Health Development, Mahidol University

[5] Department of Surgery, Faculty of Medicine, Chiang Mai University

[6] NCD Centre of Excellence, Research Institute for Health Sciences, Chiang Mai University

[7] Department of Disease Control, Ministry of Public Health

[8] Department of Social Medicine, Lampang Hospital

[9] Faculty of Nursing, Suratthanni Rajabhat University

[10] Faculty of Public Health and Policy, London School of Hygiene and Tropical Medicine

[11] Diabetes Research Center, University of Leicester

Word count: 3769 excluding references

Corresponding author: Chaisiri Angkurawaranon, Department of Family Medicine, Faculty of Medicine, Chiang Mai University, email: chaisiri.a@cmu.ac.th

For peer review only - http://bmjopen.bmj.com/site/about/guidelines.xhtml

ABSTRACT

Introduction Type 2 diabetes mellitus is amongst the foremost health challenges facing policy makers in Thailand as its prevalence has more than tripled over the last two decades, accounting for considerable death, disability and healthcare expenditure. Diabetes Self-Management Education (DSME) programmes show promise in improving diabetes outcomes, but this is not routinely utilised in Thailand. This study aims to test a culturally-tailored DSME model in Thailand, using a 3-arm cluster randomised controlled trial comparing a nurse-led model, a peer-assisted model, and standard care. We will test which model is effective and cost effective to improve cardiovascular risk and control of blood glucose among people with diabetes.

Methods and analysis 21 primary care units in northern Thailand will be randomised to one of three interventions, enrolling a total of 693 patients. The primary care units will be randomised (1:1:1) to participate in a culturally-tailored DSME intervention for 12 months. The three-arm trial design will compare effectiveness of nurse-led, peer-assisted (Thai village health volunteers) and standard care. The primary trial outcomes are changes in haemoglobin A1c and cardiovascular risk score. A process evaluation and cost effectiveness evaluation will be conducted to produce policy relevant guidance for the Thai Ministry of Public Health. The planned trial period will start in January 2020 and finish October 2021.

Ethics and dissemination Ethical approval has been obtained from Thailand and the UK. We will share our study data with other researchers, advertising via our publications and web presence. In particular, we are committed to sharing our findings and data with academic audiences in Thailand and other low- and middle-income countries.

For peer review only

Trial registration number:

ClinicalTrials.gov ID NCT03938233

Strengths and limitation of this study

- A three-arm cluster randomized controlled trial to evaluate clinical and cost-effectiveness of a culturally tailored DSME under two alternative modes of delivery (nurse-led and peer-assisted) will provide policy makers with options for scalability.
- A culturally-tailored DSME programme has been developed with input from stakeholders (policy makers, clinicians, nurses, village health volunteers and people with diabetes).
- A series of short films has been developed to introduce key topics, as there is increasing recognition that films are a highly efficient medium for communicating information, particularly in low literacy settings

For peer review only

For peer review only - http://bmjopen.bmj.com/site/about/guidelines.xhtml

INTRODUCTION

Type 2 diabetes mellitus (hereto referred to as diabetes) is amongst the foremost health challenges facing policy makers in Thailand. Its prevalence has more than tripled over the last two decades to an estimated 4 million adults (age adjusted prevalence 7.1%) living with diabetes in 2015.[1,2] Diabetes is associated with several macrovascular (e.g. ischaemic heart disease) and microvascular complications (e.g. nephropathy, retinopathy, neuropathy, and foot disease), which primarily account for the considerable death and disability (of which diabetes is the 5th leading cause in Thailand)[3]. In addition, diabetes in Thailand causes a two-fold increase in healthcare expenditure and significant loss of economic productivity—of both people with diabetes and their carers.[1]

The complications of diabetes can be largely prevented or delayed through lifestyle change and medication when necessary, and regular screening for early detection and management of complications to control risk factors such as blood glucose, lipids and blood pressure.[4,5] Under Thailand's universal health coverage, nearly everyone diagnosed with diabetes receives timely medical care (>97%) and has access to screening. Yet, surveys suggest that only about half of the people with diabetes achieve optimal control of risk factors or receive annual screening for microvascular complications (53-60%).[1,6] Limited data support a lack of engagement and self-management skills among those diagnosed with diabetes as the main underlying reasons for this.[7] Successful management of diabetes involves a considerable degree of self-management. People with diabetes need to adhere to multiple behaviours, including healthy lifestyles, regular monitoring and medication, problem-solving and healthy coping strategies. In this, they are greatly supported by diabetes self-management education (DSME), defined as 'a collaborative and ongoing process intended to facilitate the development of knowledge, skills, and abilities that are required for successful self-management of diabetes'.[8] Evidence from over 100 studies,

For peer review only

For peer review only - http://bmjopen.bmj.com/site/about/guidelines.xhtml

including many randomised controlled trials conducted predominantly in high-income countries, suggests that DSME programmes are associated with improvements in a range of behavioural outcomes (knowledge, behaviours, self-efficacy, psychosocial), and clinical outcomes (physiological risk factors, screening for complications, quality of life),[9,10] and are-cost-effective.[11] Therefore, DSME programmes are recommended by most clinical guidelines.[8] However, there is considerable heterogeneity in the effectiveness of DSME programmes[9,10]. Programmes that are more effective usually offer more than 10 hours of contact between trainers and patients, incorporate behavioural approaches and provide longer-term support mechanisms. However, providing intensive and sustained support has cost implications, resulting in ongoing efforts to identify more cost-efficient ways to deliver DSME, notably through use of lay health workers or peer educators, such as Thai village health volunteers (VHV).

Peers can support sustained changes in complex health behaviours by providing assistance in daily management, social and emotional support, linkage to clinical care, and ongoing availability of support.[12,13] Unlike the educational/psychological framework of professional support, peer support operates on a social support framework. Although traditionally restricted to those with experience of disease, the definition of peers has been expanded to include other non-professionals with a close relationship with the community (e.g. VHV).[14] However, despite widespread interest, empirical data on effectiveness of peers in supporting behaviour change in chronic diseases, including diabetes, is limited and inconsistent.[15,16] In an earlier review, the World Health Organisation did not find sufficient evidence to recommend peer support programmes as a policy option for diabetes management in LMICs.[17] Whereas many studies on the effectiveness of DSME programmes come from high-income countries (HIC), there is a dearth of data from LMIC settings on cost-effectiveness, acceptability and potential adverse

For peer review only - http://bmjopen.bmj.com/site/about/guidelines.xhtml

consequences of peer support programmes, as well as optimal strategies for mobilising and integrating peers in diabetes care pathways.[13,18-19]

In the Thai healthcare system, structured DSME is not routinely available. While several small-scale studies from Thailand have demonstrated that DSME can strengthen self-management of diabetes, negative perceptions of educational programmes and concerns about the burden on existing staff time and costs, have so far prevented the introduction of DSME.[1,19] However, recent policy developments in Thailand are supportive of DSME introduction, if a scalable model can be found. We therefore hypothesise that a nurse-led and/or peer-assisted model for DSME delivery will be effective in improving blood glucose among people with diabetes, with the peer-assisted model being the more scalable option for the Thai healthcare system. We propose to evaluate this through a three-arm cluster randomised controlled trial.

METHODS AND ANALYSIS

Study design

This study is an MRC complex intervention,[20] three-arm cluster randomised controlled trial. Primary care units from within two provinces: Chiang Mai and Lampang will be randomised for patients to receive either the nurse-led or peer-assisted DSME intervention or standard care (brief education session by a nurse). Assessments will be undertaken at baseline, 6- and 12-month follow up. A process and cost-effective evaluation will also be conducted.

Setting and Participant Selection

Potential participants requiring a DSME intervention will be recruited from 21 primary care units in Chiang Mai (7 primary care units) and Lampang provinces (14 primary care units) in northern

For peer review only - http://bmjopen.bmj.com/site/about/guidelines.xhtml

Thailand. Chiang Mai is a province of over 1.4 million people with 24 district hospitals and about 250 primary care units. Lampang is a province of approximately 700,000 people with 12 district hospitals and about 140 primary care units. While diabetes is diagnosed at tertiary and district hospitals, it is managed at the primary care unit health centres, which are served by a full-time nurse (doctor visits weekly), and 10-15 village health volunteers (VHV) linking patients in the community.

From clinical records, we will recruit all new referrals for diabetes management and patients with uncontrolled diabetes diagnosed in the past 3 years at the 21 primary care units over a 9-month period (N=693). Posters and information sheets will be used to provide necessary, trial-related information to prospective participants (Figure 1).

Participants presenting to one of the 21 primary care units will be included if they are:

1. Over 18 years of age with a new referral for type 2 diabetes management;
2. Over 18 years of age with uncontrolled diabetes (HbA1c>7 %) within the first three years of diagnosis;
2. Willing and able to attend educational group meetings; and
3. Available for 6- and 12-month follow-up visits

Participants will be excluded if they:

1. Have advanced diabetic complications such as diabetic nephropathy, diabetic retinopathy, or amputations; or if they are pregnant as patients with these conditions and co-morbidities are usually referred to secondary care facilities for treatment in Thailand and not often managed in primary care where this trial is conducted.
2. Have learning disabilities, dementia or active severe mental illness; or

For peer review only

For peer review only - http://bmjopen.bmj.com/site/about/guidelines.xhtml

3. Lack the capacity to give voluntary, informed consent.

Randomisation

Stratified by province, 21 primary care units (7 from Chiang Mai and 14 from Lampang) will be randomised to provide one of three interventions: (1) nurse-led DSME; (2) nurse-led DSME with peer assistance (provided by Thai village health volunteers, VHV); or (3) standard care (brief education session by a nurse), resulting in seven primary care units in each arm of the study. All primary care units follow protocols for diabetes management as outlined by national guidelines. Stratification by province will minimise any variation in practice between the different primary care units.

Sample size calculation

The trial is powered to detect a difference in HbA1c of 0.6% (SD 1.5%) between control and intervention arms, based on the effect size of 0.6% noted in a previous diabetes management study in Thailand[21], and the fact that an increase in HbA1c of ~0.5% was associated with increased mortality among people with diabetes[22]. An ICC between primary care units of 0.02 was assumed based on a similar study which found that the intraclass correlation for HbA1c at three years was 0.02 (95% confidence interval 0.00 to 0.08)[23]. Allowing for a loss-to-follow up rate of 20%, 693 participants are needed from 21 primary care units (7 in each trial arm arm) to achieve 80% power at 2.5% significance level.

Informed consent

For peer review only - http://bmjopen.bmj.com/site/about/guidelines.xhtml

Written informed consent will be obtained from all study participants in Thai before any study procedures are undertaken including enrolment, intervention allocation, follow-up interviews and blood draws. Local research assistants will explain the study to patients using the patient information sheet (supplementary file). The right of the patient to refuse to participate without giving reasons will be respected.

Intervention description and delivery

The DSME programme has been developed using a structured process. This included a desk and literature review and focus groups with local nurses and VHV to develop a paper prototype of the intervention, including a hypothesised pathway of action (e.g. common-sense model of illness, empowerment, discovery learning, social learning, and social support) and a training manual for nurses and VHV. In addition, seven brief films (5-6 minutes long) have been developed and have been used to trigger discussions on key topic areas during DSME intervention meetings covering such topics as medical adherence, dietary recommendation, physical activity and stress management. Films will be used in the intervention as they are increasingly recognised as a highly effective medium for improved recall when communicating large amounts of information, particularly in low literacy settings.[24] Films will be in the local language and use local people.

Our DSME programme will consist of four modules. Module 1 covers the general overview of diabetes, treatment targets and goal setting. Module 2 covers diet and nutrition. Module 3 covers physical activity and exercise, while module 4 covers stress management and mental health.

For peer review only - http://bmjopen.bmj.com/site/about/guidelines.xhtml

Each module takes approximately 1.5 hours. Each participant is given an information and self-assessment booklet which covers all content and materials for the four modules.

In addition to routine care, in the nurse-led arm, the nurse will deliver the DSME to groups of 5-10 participants per session within the first months after enrolment. The participants in the nurse-led arm will also be given a refresher session going over all 4 modules again at 6 months after enrolment. For the peer-assisted arm, a VHV will participate as an assistant to the nurse in the first DSME session. However, the VHV will lead the refresher course at 6 months. In addition, participants in the peer-assisted arm will receive monthly contact with the VHV either via a home visit or telephone call. During these brief 15-20 minute monthly contacts, VHV will ask about the progress made, providing encouragement if plans for self-management are being followed or discussing ways to overcome barriers and set new goals if obstacles are identified. Contents of the three trial arms are summarized in Table 1.

For peer review only

Table 1: Summary of DSME delivery in the three trial arms

Month	Routine care	Nurse-led DSME	Peer-assisted DSME*
0	Individual session	Nurse provides DSME (4 modules)	Nurse provides DSME (4 modules) with VHV to assist the sessions
6	Individual session	Refresher course (4 modules) provided by nurse	Refresher course (4 modules) led by VHV
12	Outcome assessment	Outcome assessment	Outcome assessment

* participants in the peer-assistant arm will additionally receive monthly contact with the VHV either via a home visit or telephone call.

The intervention will be piloted at four community primary care units with four nurses and four VHV who will be trained to deliver the DSME programme to groups of 5-10 persons with diabetes. This will allow for refining the intervention, ensuring data collection can be completed as specified, and to check our assumptions and processes for the trial. For the main trial, a two-day workshop will be held such that at least one nurse and one VHV from each primary care unit will be trained by the Thai research team to deliver the DSME programme at the community primary care unit or neighbourhoods as appropriate. The trial coordinator will conduct periodic site visits as additional training as requested and a line of communication will be established between the research team and each site to answer any issues which may arise.

For peer review only - http://bmjopen.bmj.com/site/about/guidelines.xhtml

A process evaluation using qualitative methods will be conducted during the trial period and at the end of the study. Observations including video recordings of intervention delivery will be made. We plan to conduct 5-10 focus groups among providers (nurses and VHV) to explore healthcare professionals' perspectives regarding their experience and implementation of the DSME programme, including views on the cultural transferability of DSME and scalability to the Thai context. In addition, we plan to conduct 20 structured interviews with patients. These evaluations will help assess intervention delivery (fidelity, dose and reach), clarify causal mechanisms (those hypothesised by theory of change developed within the project or emergent mechanisms identified), and detail contextual factors (barriers, facilitators) associated with variation in outcomes.[25] The process evaluation will also consist of one-to-one interviews with clinicians and policy makers and direct observations of patients. Data for economic evaluations (resource usage and quality of life using EQ-5D[26]) will be obtained prospectively alongside the trial.

Standard care

Patients in the control group will receive standard care in the form of a brief didactic educational session at the time of diagnosis of diabetes and during routine clinic visits at 6 months.[27]

Study outcomes

The two primary outcomes of the intervention are a difference in trial arms at one-year follow-up in HbA1c and cardiovascular risk score. There is a growing recognition of the importance of combining tight glycaemic control with reduction in other cardiovascular risk factors for prevention of or reduction in complications.[5] The cardiovascular risk will be estimated by the

For peer review only - http://bmjopen.bmj.com/site/about/guidelines.xhtml

Thai cardiovascular risk score model, which estimates the risk of dying from any cardiovascular disease over 10 years based on age, gender, smoking habits, total cholesterol and systolic blood pressure, as it has been calibrated for use in a Thai population.[28]

Additional secondary outcomes include changes at one year for biological, physical, psychosocial, lifestyle and intervention-related measures, as described in Table 2.

Table 2: Summary of primary and secondary outcome measures

**Primary outcomes**	**Measures or questionnaires**
Haemoglobin A1c levels (HbA1c)	HbA1c will measure the average blood glucose (sugar) levels over the past two to three months
Thai Cardiovascular risk score	Estimates the risk of dying from any cardiovascular disease over 10 years based on age, gender, smoking habits, total cholesterol and systolic blood pressure.
**Secondary outcomes**	**Measures or questionnaires**
Biological and physical measures	Body weight, Body Mass Index; BMI, Blood lipids (Total, LDL-C HDL, triglycerides), waist circumference, blood pressure, fasting blood glucose
Quality of life	WHOQOL-BREF. A 26 item questionnaire developed by WHO to assess quality of life in adults[29]
	The European Quality of life questionnaire; EuroQol EQ-5D 5L[26] EQ-5D is a quality of life measure that includes five quality of life questions on mobility, self-care, usual activity, pain,

For peer review only

	anxiety/depression and a scale of 0 to 100 on how the person is feeling on that day.
Depression	Hospital Anxiety and Depression Scale; HADS[30]<br>HADS measures depression and anxiety that will address psychological change with a scale from 0 to 3.
Stress	Perceived Stress Questionnaire; PSS[31]<br>PSS is a psychological instrument for measuring the perception of stress. Ten items with a scale from 1 to 4.
Physical activity	International Physical Activity Questionnaire; IPAQ[32]<br>Short form IPAQ is an assessment of physical activity comprising of seven questions. There are two forms of output from scoring the IPAQ. Results can be reported in categories (low activity levels, moderate activity levels or high activity levels) or as a continuous variable (MET-minutes a week). MET-minutes represent the amount of energy expended carrying out physical activity.
Diabetes knowledge and skills	Brief diabetes Illness Perception Questionnaire; B-IPQ[33].<br>B-IPQ has nine components of which the first five questions assess the cognitive representation of illness perception, two of the questions assess the emotional representation, one item assesses comprehensibility and one item on the root cause of the illness
	Diabetes Self-Management Education and Support; DMSES[34].<br>DMSES is one of the most widely used scale in measuring self-efficacy in type 2 diabetes management. The Thai-DMSES has

For peer review only - http://bmjopen.bmj.com/site/about/guidelines.xhtml

	twenty questions which has been demonstrated to have good psychometric properties[35]
	Summary of Diabetes Self-Care Activities questionnaire SDSCA[36] SDSCA is a diabetes self-care activities questionnaire focusing on general diet, diabetes-specific diet, physical activity, blood-glucose testing, foot care, and smoking.
Satisfaction with intervention	Chronic Illness Resources Survey (CIRS)[37] CIRS is a questionnaire to represent patient’s received support. Individual’s support for behavioural-specific disease management is assessed: proximal support e.g. friend and family and distal factor e.g. neighbourhood or community.
	Modified Medical Interview Satisfaction Scale (MISS-21)[38] MISS-21 is a questionnaire to measure patient satisfaction with patient and health care professional communication/consultation.

For peer review only

Data collection and follow-up

Each participant will be involved in the study for 12 months after taking consent and baseline data. The trial is expected to start January 2020 and finish October 2021.

Data collection methods will include:

a) Questionnaires: Questionnaire data will be collected face-to-face by research assistants for the full sample at baseline, 6 and 12 months at the community primary care unit where participants are recruited. A custom-designed form linked to RedCap will be used to collect, validate, verify,

For peer review only - http://bmjopen.bmj.com/site/about/guidelines.xhtml

and store respondents' data where possible or else data will be collected via paper forms and double-entered into the databases. All data files and databases will be password protected.

b) Biological samples: Blood samples will be collected at baseline, 6 and 12 months, to measure fasting blood glucose, HbA1c and lipids, coordinating where possible with the annual routine tests offered to patients to reduce duplication. All blood samples will be administered from participants by trained phlebotomists. Data will be linked to the participant information using a unique respondent ID, which will be assigned to all study participants.

c) Interviews: During the delivery of the intervention, a process evaluation using a subset of participants will be conducted using in-depth interviews and focus group discussions. These will be audio-recorded. Data will be collected using a range of qualitative methods: a) one-to-one interviews, and focus group discussions with nurses, health volunteers, people with diabetes and their carers (5-10 focus groups, and 20 semi-structured interviews) and b) ethnography through direct observations including video recordings of intervention delivery, and unstructured interviews with clinical managers and policy makers. The data collected will be used to capture the range of experiences of the intervention, and identify unanticipated pathways to generate new theories as well as exploring the scalability of the intervention.

Trial follow-up appointments

The research team will hold weekly briefings with the study coordinators to generate a list of priority areas and loss to follow-up participant lists. Arrangements to follow-up participants who have not turned up for their appointment will be made, with attempts to contact participants

For peer review only - http://bmjopen.bmj.com/site/about/guidelines.xhtml

through SMS, phone calls, or house visits. Participants will be declared lost to follow-up if they do not show for a month and are untraceable.

Data management

A data collection protocol will be developed, and the study coordinator in Thailand will provide training to fieldworkers before data collection commences. Validation will be performed on a random sample of questionnaire data by crosschecking with clinic records. Any discrepancies will be followed-up and addressed by field workers, re-contacting participants to clarify as necessary. Using Redcap (https://redcap.med.cmu.ac.th), quantitative data will be entered directly via a form with built-in data checks to minimise transcription errors (or where necessary collected on paper and later double entered into the electronic form). Post entry checks will be conducted by exploring the distribution, ranges, and outliers of each variable. All hospital laboratories have their own internal quality assurance protocols and are also linked to a national external quality assurance mechanism. Fieldworkers will be trained in qualitative methods and an interview schedule will be devised.

Statistical analysis

1. Quantitative analysis

Available outcome data will be analysed on an intention-to-treat basis. Potential clustering of outcomes (HbA1c at 12 months and CVD risk score at 12 months) at the level of community primary care units will be accounted for using random intercept models. To improve precision of the estimates, outcomes will be adjusted for their baseline values. In case of baseline imbalances

For peer review only - http://bmjopen.bmj.com/site/about/guidelines.xhtml

For peer review only

of relevant covariates (e.g. age, education level, body mass index), judged by statistical significance at $p<0.05$, we will conduct a secondary analysis adjusting for these covariates.

2. Qualitative analysis

Qualitative data from interviews, focus groups and direct observations be will transcribed and analysed using NVivo software. The data will be analysed using a descriptive, phenomenological approach to understand participants' experiences and interpret them within their respective cultural contexts. Comparative analysis will compare and contrast these themes across participants. Deviant cases will be actively sought throughout the analysis and emerging ideas and themes modified in response. In addition, thematic analysis will be used to inform elements of scalability and to produce a set of considerations in making decisions about the scalability of the intervention.

3. Cost-effective analysis

Data for economic evaluation (resource usage and quality of life using EQ-5D) will be obtained prospectively alongside the trial. We will aim to capture all health service contacts, as well as out-of-pocket expenses and medication use. Educator training costs will be included, as well as minimal intervention material costs (as most will be made available freely after the trial). Utility values from EQ-5D will be derived using a Thai tariff. Incremental cost utility will be estimated from the Thai health system and societal perspectives, to provide incremental cost-effectiveness ratio and probability of being cost-effective at Thai government's willingness to pay threshold of 160,000 baht/QALY.

For peer review only - http://bmjopen.bmj.com/site/about/guidelines.xhtml

For peer review only

ETHICS AND DISSEMINATION

This study is to be conducted according to the international standards of Good Clinical Practice (International Conference on Harmonization guidelines), Declaration of Helsinki, and International Ethical Guidelines for Biomedical Research Involving Human Subjects, applicable national government regulations, and institutional research policies and procedures. All investigators received GCP training at the onset of the study. Ethical approval was obtained prior to commencement of the project from Chiang Mai University [No 326/2018] and the London School of Hygiene & Tropical Medicine [16113/RR/12850]. The study protocol, informed consent form, patient information sheet and other relevant information has been approved. Any future amendments of the protocol shall be submitted to and approved by the Institutional Review Board (IRB) before implementation.

Trial monitoring and oversight

The role of the Trial Steering Committee (TSC) is to provide overall supervision of the trial. The TSC will meet every six months. The TSC will include experts in the field of DSME, health psychology and clinical trials, as well as an independent Chair.

Dissemination

Research findings will be disseminated to scientific audiences at major conferences and published in high-impact, open-access scientific journals; planned publications include those on

For peer review only - http://bmjopen.bmj.com/site/about/guidelines.xhtml

intervention development, primary trial results, process evaluation, and health systems analysis, at a minimum. This study is expected to have a major policy impact due to the close involvement of a key policy maker in the project. Towards the end of the study a dedicated workshop will be held with key governmental stakeholders to disseminate the recommended model for DSME implementation in Thailand and encourage inclusion of a large-scale scientific evaluation into any national implementation of the scheme.

Contributors: CA, SS, OQ, PACM, SK were involved in conception and trial design. CA, KP, PACM and SK wrote the first draft of the trial design proposal. KR, WT, KH, KK were involved in critical revision of the trial design proposal. All authors contributed to finalising the trial protocol. CA, KP, IPN, CP, AH, NW were involved with preparation of the manuscript for submission. All authors reviewed the manuscript and approved the manuscript for publication.

Funding

This study is supported by UK Medical Research Council (MRC) grant number [MR/R020876/1] and the Thailand Research Fund (TRF) grant number [DBG6180007].

Data availability

Data will be shared upon reasonable request

Patient and Public Involvement

Patients or the public WERE NOT involved in the design, or conduct, or reporting, or dissemination plans of our research.

For peer review only

Trial registration and version

The trial has been registered (ClinicalTrials.gov ID NCT03938233 Version 3 last update January 18,2020)

Trial sponsor

This study is sponsored by London School of Hygiene & Tropical Medicine, Keppel Street, London WC1E 7HT, UK [16113].

Competing interests

None declared

Ethics approval

The study has been approved by the Chiang Mai University Research Ethics Committee [326/2018] and the London School of Hygiene & Tropical Medicine [16113/RR/12850].

Figure 1 Legend: Trial flow diagram

REFERENCES

1. Deerochanawong C, Ferrario A. Diabetes management in Thailand: a literature review of the burden, costs, and outcomes. Glob Health 2013; 9: 11.
2. International Diabetes Federation. IDF Diabetes Atlas Seventh Edition. 2015.
3. Jiraporncharoen W, Pinyopornpanish K, Junjom K, et al. Exploring perceptions, attitudes and beliefs of Thai patients with type 2 diabetes mellitus as they relate to medication adherence at an out-patient primary care clinic in Chiang Mai, Thailand. BMC Family Practice 2020;21(1):173. doi: 10.1186/s12875-020-01233-7

For peer review only

4. Effect of intensive blood-glucose control with metformin on complications in overweight patients with type 2 diabetes (UKPDS 34). The Lancet 1998; 352: 854–65.

5. Gaede P, Vedel P, Larsen N, Jensen GVH, Parving H-H, Pedersen O. Multifactorial intervention and cardiovascular disease in patients with type 2 diabetes. N Engl J Med 2003; 348: 383–93.

6. Rangsin R. An assessment of quality of care among patients diagnosed with type 2 diabetes and hypertension visiting hospitals of ministry of public health and Bangkok Metropolitan Administration in Thailand. 2012.

7. Auemaneekul N, Chandanasotthi P, Rawiworrakul T, et al. Community partnership, context-based intervention and diabetes control in Thailand. J Public Health 2016; 46: 236–47.

8. Haas L, Maryniuk M, Beck J, et al. National standards for diabetes self-management education and support. Diabetes Educ 2012; 38: 619–629.

9. Chrvala CA, Sherr D, Lipman RD. Diabetes self-management education for adults with type 2 diabetes mellitus: A systematic review of the effect on glycemic control. Patient Educ Couns 2016; 99: 926–43.

10. Pillay J, Armstrong MJ, Butalia S, et al. Behavioral programmes for type 2 diabetes mellitus: A systematic review and network meta-analysis. Ann Intern Med 2015; 163: 848.

11. Doyle G, O'Donnell S, Quigley E, et al. Patient level cost of diabetes self-management education programmes: an international evaluation. BMJ Open 2017; 7: e013805.

For peer review only

For peer review only - http://bmjopen.bmj.com/site/about/guidelines.xhtml

12. Fisher EB, Boothroyd RI, Elstad EA, et al. Peer support of complex health behaviors in prevention and disease management with special reference to diabetes: Systematic reviews. Clin Diabetes Endocrinol 2017; 3. DOI:10.1186/s40842-017-0042-3.

13. Dale JR, Williams SM, Bowyer V. What is the effect of peer support on diabetes outcomes in adults? A systematic review. Diabet Med 2012; 29: 1361–77.

14. Norris SL, Chowdhury FM, Van Le K, et al. Effectiveness of community health workers in the care of persons with diabetes. Diabet Med J Br Diabet Assoc 2006; 23: 544–56.

15. World Health Organization. Peer support programmes in diabetes: report of a WHO consultation 5-7 November 2007. Geneva, Switzerland: WHO, 2008.

16. Fisher EB, Boothroyd RI, Coufal MM, et al. Peer support for self-management of diabetes improved outcomes in international settings. Health Aff (Millwood) 2012; 31: 130–9.

17. Trento M, Gamba S, Gentile L, et al. Rethink Organization to iMprove Education and Outcomes (ROMEO): A multicenter randomized trial of lifestyle intervention by group care to manage type 2 diabetes. Diabetes Care 2010; 33: 745–7.

18. Werfalli M, Raubenheimer P, Engel M, et al. Effectiveness of community-based peer-led diabetes self-management programme (COMP-DSMP) for improving clinical outcomes and quality of life of adults with diabetes in primary care settings in low and middle-income countries (LMIC): A systematic review and meta-analysis. BMJ Open 2015; 5: e007635.

For peer review only - http://bmjopen.bmj.com/site/about/guidelines.xhtml

19. Keeratiyutawong P, Hanucharurnkl S, Melkus G, Vorapongsathon OPT. Effectiveness of a self-management programme for Thais with type 2 diabetes. Thai J Nurs Res 2006; 10: 85–97.

20. Craig P, Dieppe P, Macintyre S, Michie S, Nazareth I, Petticrew M. Developing and evaluating complex interventions: the new Medical Research Council guidance. BMJ 2008; a1655.

21. Wattana C, Srisuphan W, Pothiban L, Upchurch SL. Effects of a diabetes self-management programme on glycaemic control, coronary heart disease risk, and quality of life among Thai patients with type 2 diabetes. Nurs Health Sci 2007; 9: 135–41.

22. Wan EYF, Fung CSC, Wong CKH, Chin WY, Lam CLK. Association of hemogloblin A1c levels with cardiovascular disease and mortality in Chinese patients with diabetes

23. Khunti K, Gray LJ, Skinner T, et al. Effectiveness of a diabetes education and self-management programme (DESMOND) for people with newly diagnosed type 2 diabetes mellitus: Three-year follow-up of a cluster randomised controlled trial in primary care. BMJ 2012; 344: e2333–e2333.

24. Tuong W, Larsen ER, Armstrong AW. Videos to influence: A systematic review of effectiveness of video-based education in modifying health behaviors. J Behav Med 2014; 37: 218–33.

25. Moore GF, Audrey S, Barker M, et al. Process evaluation of complex interventions: Medical Research Council guidance. BMJ 2015; 350: h1258–h1258.

For peer review only

26. Kimman M, et al. Validity of the Thai EQ-5D in an occupational population in Thailand. *Qual Life Res* 2013 Aug; 22(6):1499-506.

27. Clinical Practice Guidelines for Diabetes. Diabetes Association of Thailand, 2017. (https://www.dmthai.org/index.php/knowledge/healthcare-providers/cpg/443-guideline-diabetes-care-2017)

28. Aekplakorn A, Bunnag P, Woodward M. et al. Risk score for predicting incident diabetes in the Thai population. *Diabetes Care* 2006 Aug; 29(8):1872-1877

29. Mahatnirunkul S. Tuntipivatanaskul W, Pumpisanchai W, et al. Comparison of the WHOQOL-100 and the WHOQOL-BREF. *Journal of Mental Health of Thailand* 1998; 5: 4-15.

30. Nilchaikovit T. et al. Development of Thai version of hospital anxiety and depression scale in cancer patients. *J Psychiatr Assoc Thailand* 1996; 41(1):18-30.

31. Wongpakaran N, Wongpakaran T. The Thai version of the PSS-10: An investigation of its psychometric properties. *BioPsychoSocial Medicine* 2010: 4:6

32. Rattanawiwatpong P, et al. Validity and reliability of the Thai version of Short Format International Physical Activity Questionnaire (IPAQ). *J Thai Rehabil* 2006; 16(3):147-160.

33. Leelacharas S, et al. Illness perceptions, lifestyle behaviours, social support and cardiovascular risks in people with Hypertension in Urban and Rural Areas of Thailand. Pacific Rim Int J Nur Res 2015; 19

34. Lamsumang, W. The development of the Thai version of the diabetes management self-efficacy scale (T-DMSES) for older adults with type 2 diabetes. State University of New York at Buffalo, New York; 2009.

For peer review only - http://bmjopen.bmj.com/site/about/guidelines.xhtml

35. Sangruangake M, Jiraporkul C, Hurst C. Psychometric properties of diabetes management self-efficacy in Thai type 2 diabetes mellitus patients: A multicenter study. *Int J Endocrinol*. 2017;2017:2503156. doi:10.1155/2017/2503156)

36. Keeratiyatawong, P, Hanucharumkul, S., Melkus, G. D. E., Panpakdee, O., Vorapongsathorn T. Effectiveness of a self-management programme for Thais with type 2 diabetes. Thai J Nurse Res. 2006; 10: 85-97

37. Manit A, et al. Development of needs and resources for self-management assessment instrument in Thais with type 2 diabetes: Cross-cultural adaptation. *J Med Assoc Thai* 2011; Vol. 94 No. 11.

38. Meakin R and Weinman J. The 'Medical Interview Satisfaction Scale' (MISS-21) adapted for British general practice. *Family Practice* 2002; 19:257–263.

For peer review only - http://bmjopen.bmj.com/site/about/guidelines.xhtml

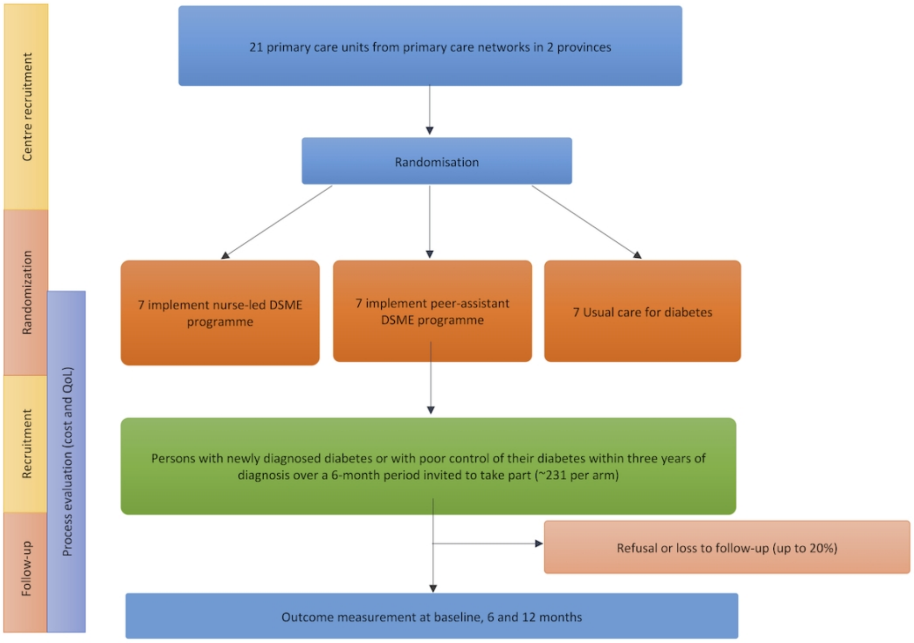

Figure 1: Trial flow diagram

27x20mm (1200 x 1200 DPI)

CHIANG MAI UNIVERSITY

LONDON SCHOOL of HYGIENE &TROPICAL MEDICINE

Participant Information Sheet

Title of Project: Delivering Diabetes Self-Management Education in Thailand

Introduction

We would like to invite you to take part in a research study. Joining the study is entirely up to you. Before you decide, you need to understand why the research is being done and what it would involve. One of our team will go through this information sheet with you, and answer any questions you may have. Ask questions if anything you read is not clear or you would like more information. Please feel free to talk to others about the study if you wish. Take time to decide whether or not to take part.

What is the purpose of the study?

The London School of Hygiene and Tropical Medicine (LSHTM) are conducting research for people with type 2 diabetes in Chiang Mai Thailand who have recently been diagnosed. The purpose of this study is to provide diabetes self-management education (DSME) by trained nurses and peer health care volunteers for those recently diagnosed.

This is a 3 arm cluster randomised trial where your service provider has been randomised to one of the following 3 interventions: 1) usual treatment 2) Nurse led DSME 3) Peer led DSME.

Why have I been asked to take part?

You have been invited because your healthcare provider is involved in the study and has identified you as being newly diagnosed with type 2 diabetes.

Do I have to take part?

No. It is up to you to decide to take part or now. If you don't want to take part, that's ok. Your doctor will still care for you and your decision will not affect the quality of care you receive.

We will discuss the study together and give you a copy of this information sheet. If you agree to take part, we will then ask you to sign a consent form.

What will happen to me if I take part?

Enrolling you in the study

If you are interested in taking part the research assistant will go through the information sheet with you. If you are happy with the information provided you may complete the consent form.

At this point you will also have the opportunity to raise with the researcher any questions you might have about the study. You do not have to enter the study unless you feel completely happy with what you are being asked to do.

For peer review only

For peer review only - http://bmjopen.bmj.com/site/about/guidelines.xhtml

CHIANG MAI UNIVERSITY

LONDON SCHOOL of HYGIENE &TROPICAL MEDICINE

Participating in a diabetes education programme

You will be invited to attend a diabetes self-management educational programme. This programme will consist of a number of sessions delivered as monthly meetings led by community health volunteers or nurses. Short films about living with diabetes will be shown to introduce key topics.

The education and skills training will be followed by an open discussion session to discuss common challenges and solutions, set lifestyle goals and seek advice.

Collecting information

If you are happy to proceed with the study the researcher will then ask a series of questions relating to your health. This will be through completing a questionnaire booklet. These baseline questions will take about twenty minutes to complete. We will also take blood samples as the beginning of the study from trained phlebotomists.

Each participant will be compensated 200 baht for each of the baseline, 6 month and 12 month visit.

Follow up

Six months and twelve months after you have entered the study we will contact you again to ask you a further set of questions and blood samples to see how you are feeling now.

How is taking part in the study different from usual care?

Additional support and education may be provided through the study through the self-management education programme.

Whilst you are taking part in the study you will continue to be looked after by your healthcare providers, as normal. No treatment will be withheld from you during the course of this study.

The researcher will want you to complete some questionnaires at the beginning of the study and after six and twelve months. You may be asked to take part in an interview with a researcher.

What are the possible risks and disadvantages?

It will take some time to complete the questionnaire, around twenty minutes to complete. If you agree to be further interviewed, then this will also mean time will be needed to attend the interview. You may find minor discomfort from the taking blood samples. We will have trained phlebotomists to take the blood sample.

What are the possible benefits?

We cannot promise the study will help you but the information we get from the study will help our knowledge and understanding of this research area within diabetes self-management education in Thailand.

For peer review only - http://bmjopen.bmj.com/site/about/guidelines.xhtml

For peer review only

CHIANG MAI UNIVERSITY

LONDON SCHOOL of HYGIENE &TROPICAL MEDICINE

What if something goes wrong?

If you have a concern about any aspect of this study, you should ask to speak to the researchers who will do their best to answer your questions <+66(0)616852307>. If you remain unhappy and wish to complain formally, you can do this by contacting <if LSHTM is the sponsor: Patricia Henley at rgio@lshtm.ac.uk or +44 (0) 20 7927 2626>

The London School of Hygiene and Tropical Medicine holds insurance policies which apply to this study. If you experience harm or injury as a result of taking part in this study, you may be eligible to claim compensation.

Can I change my mind about taking part?

Yes. You can withdraw from the study at any time. You just need to tell your doctor that you don't want to be in the study anymore. Your doctor will still care for you.

If you withdraw from the study we will destroy all your identifiable samples/ tape recorded interviews, but we will need to use the data collected on you up to your withdrawal

Or

You can withdraw from treatment but keep in contact with us to let us know your progress. Information collected may still be used. Any stored blood or tissue samples that can still be identified as yours will be destroyed if you wish, or will continue to be stored for further research.

What will happen to information collected about me?

All information collected about you will be kept private. Only the study staff and authorities who check that the study is being carried out properly will be allowed to look at information about you. Data may be sent to other study staff in London or in Chiang Mai but this will be anonymised. This means that any information about you which leaves the hospital, will have your name and address removed so that you cannot be recognised.

Your doctor will send some details about you to the study team in Chiang Mai University who will store it securely. Your personal details will be kept in a different safe place to the other study information and will be destroyed within 10 years of the end of the study.

At the end of the project, the study data will be archived at Chiang Mai University. The data will be made available to other researchers worldwide for research and to improve medical knowledge and patient care. Your personal information will not be included and there is no way that you can be identified.

What will happen to the results of this study?

The study results will be published in a medical journal so that other doctors can learn from them. Your personal information will not be included in the study report and there is no way that you can be identified from it.

Who is organising and funding this study?

London School of Hygiene & Tropical Medicine is the sponsor for the research and they have full responsibility for the project including the collection, storage and analysis of your data.
This study is funded by the Medical Research Council UK and The Thailand Research Fund

Who has checked this study?

All research involving human participants is looked at by an independent group of people, called a Research Ethics Committee, to protect your interests. This study has been reviewed and given favourable opinion by The London

For peer review only - http://bmjopen.bmj.com/site/about/guidelines.xhtml

School of Hygiene and Tropical Medicine Research Ethics Committee (<ref: 16113>). The Research Ethics Committee, Faculty of Medicine, Chiang Mai University (No 326/2018) has also reviewed the study and have agreed that it is okay for us to ask people to take part.

Further information and contact details

Thank you for taking time to read this information leaflet. If you think you will take part in the study please read and sign the consent form.

If you would like any further information, please contact Dr. Chaisiri Angkurawaranon, MD, who can answer any questions you may have about the study.

Contact details: Chaisiri Angkurawaranon, Department of Family Medicine, Faculty of Medicine, Chiang Mai University. Tel +66(0)616852307, Email: chaisiri.a@cmu.ac.th

What happens if new information becomes available during the study?

Sometimes during a study, new information becomes available about the treatment being studied. If this happens, the research team will tell you and discuss whether you want to continue in the study. If you decide to stop taking part in the study your usual care will continue. If you decide to continue in the study you may be asked to sign an updated consent form. If we think you should withdraw from the study, we will explain the reasons and arrange for your care to continue.

What happens when the study stops?

Very occasionally a study is stopped early. If this happens, the reasons will be explained to you.

Tissue studies:

What will happen to the samples I give?

Blood samples will be used to measure HbA1c and lipids, coordinating where possible with the annual routine tests offered to patients to reduce duplication. Blood will be drawn by a trained phlebotomist when participants come for interview and sent to laboratory for analysis. Data will be linked to the participant information using a unique respondent ID, which will be assigned to all study participants.

Blood samples will be sent to a laboratory within the Chiang Mai area. The results of the blood tests will be provided to your health care professional who will discuss with you if there are any issues.

We may use some of the samples collected for future studies. These will be anonymised when stored, and all future research using these samples will be reviewed by an independent ethics committee.

A copy of this informed consent document to be offered to the participant

For peer review only - http://bmjopen.bmj.com/site/about/guidelines.xhtml

CONSENT FORM FOR PARTICIPANT AND REPRESENTATIVE

Title of Project: A scalable solution for delivery of Diabetes Self-Management Education in Thailand

Name of PI/Researcher responsible for project: Chaisiri Angkurawaranon

**Statement**	**Please initial or thumbprint* each box**
I confirm that I have read and understood the information sheet dated 08.10.2018 (version 1.0) for the above named study. I have had the opportunity to consider the information, ask questions and have these answered satisfactorily.	
I understand that my consent is voluntary and that I am free to withdraw this consent at any time without giving any reason and without my medical care or legal rights being affected.	
I understand that relevant sections of my/the participant's medical notes and data collected during the study may be looked at by authorised individuals from Chiang Mai University, where it is relevant to my taking part in this research. I give permission for these individuals to have access to these records.	
I understand that data about/from me/the participant may be shared via a public data repository or by sharing directly with other researchers, and that I will not be identifiable from this information	
I understand that the tissue sample collected from me will be used to support other research in the future, and may be shared anonymously with other researchers, for their ethically-approved projects	
I give permission for a copy of this consent form, which contains my personal information, to be made available to the Trial Coordinating Centre for monitoring purposes only.	
I agree to my health care provider being informed of my participation in the study.	
I agree to me taking part in the above named study.	

Printed name of participant/Representative	Signature of participant/Representative (or thumbprint/mark if unable to sign)	Date
Printed name of person obtaining consent	Signature of person obtaining consent	Date

The participant is unable to sign. As a witness, I confirm that all the information about the trial was given and the participant/representative consented to taking part *(*only required if the participant/representative is unable to read or write)*

Printed name of impartial witness*	Signature of impartial witness*	Date

A copy of this informed consent document has been provided to the participant.

Centre Number:
Study Number:
Participant Identification Number:

For peer review only - http://bmjopen.bmj.com/site/about/guidelines.xhtml

แบบขอรับความยินยอมเข้าร่วมโครงการ (Consent ข้อมูลสำหรับผู้ป่วย)

ชื่อโครงการศึกษาวิจัย : “การพัฒนาทางออกเพื่อการขยายการบริการที่เพิ่มความสามารถในการดูแลตนเองของผู้ที่เป็นเบาหวานในประเทศไทย”

หมายเลขโครงการศึกษาวิจัย : FAM-2561-05594

ผู้ให้ทุนสนับสนุนการวิจัย : สำนักงานคณะกรรมการส่งเสริมวิทยาศาสตร์ วิจัยและนวัตกรรม (สกสว.)

แพทย์ผู้วิจัยหลัก : อ.ดร.นพ.ชัยสิริ อังกุระวรานนท์

ท่านได้รับการเชื้อเชิญให้เข้าร่วมการศึกษาวิจัยนี้เนื่องจากท่านได้รับการวินิจฉัยว่าเป็นโรคเบาหวานที่เพิ่งได้รับการวินิจฉัยมาไม่เกิน 1 ปี หรือเป็นผู้ป่วยเบาหวานที่ยังไม่สามารถควบคุมน้ำตาลได้ ขอให้ท่านกรุณาอ่านข้อมูลข้างล่างก่อน (หรือผู้วิจัยได้อ่านให้ท่านรับทราบ) หากท่านมีข้อข้องใจใดๆ เกี่ยวกับการศึกษาวิจัยนี้ และสิทธิของท่าน กรุณาซักถามจากแพทย์ผู้ทำการศึกษาวิจัย หรือ ผู้ช่วยแพทย์ที่ทำการศึกษาวิจัย ซึ่งจะเป็นผู้สามารถให้ความกระจ่างแก่ท่านได้ หากท่านตัดสินใจเข้าร่วมการศึกษาวิจัย ท่านจะได้รับเอกสารข้อมูลสำหรับผู้เข้าร่วมโครงการวิจัยและสำเนาใบยินยอมที่ท่านเซ็นชื่อกำกับเก็บไว้ 1 ฉบับ

การศึกษาวิจัยนี้เกี่ยวกับเรื่องอะไร

เนื่องจากในปัจจุบันนี้มีผู้ป่วยเป็นโรคเบาหวานจำนวนมากขึ้น และพบว่าการป่วยเป็นโรคเบาหวานนี้สัมพันธ์กับภาวะแทรกซ้อนทางสุขภาพมากมาย เพื่อให้เป็นประโยชน์ต่อการพัฒนาการดูแลรักษาผู้ป่วยเบาหวานในอนาคต ผู้วิจัยจึงสนใจที่จะศึกษาว่าการเป็นโรคเบาหวานนั้นสัมพันธ์กับการเปลี่ยนแปลงระดับชีวภาพในร่างกายอย่างไร โดยผ่านการตรวจเลือดและอุจจาระ และเพื่อศึกษาว่าการจัดทำโครงการให้ความรู้ในการดูแลตนเองของผู้ป่วยเบาหวานโดยใช้สื่อมัลติมีเดียเข้าช่วยเหลือมีผลลัพธ์ของการรักษาที่ดีขึ้นได้หรือไม่ การศึกษานี้จะรวบรวมผู้ป่วยประมาณ 600 ราย จากโรงพยาบาลชุมชนที่ให้การรักษาแบบปฐมภูมิ ในจังหวัดเชียงใหม่ และ ลำปาง

ท่านจะต้องปฏิบัติตัวอย่างไร

หากท่านตัดสินใจเข้าร่วมการศึกษาวิจัยนี้ท่านจะถูกขอร้องให้เซ็นชื่อลงในใบยินยอมท่านจะได้รับการซักประวัติเกี่ยวกับโรคประจำตัวและการรักษาที่ได้รับ ได้ตรวจร่างกาย และการเจาะเลือด

เพื่อเป็นข้อมูลเริ่มต้นก่อนการรักษา โดยทั้งนี้ท่านจะได้รับการดูแลตามปกติควบคู่ไป

จากนั้นท่านจะได้รับการอบรมเพื่อพัฒนาศักยภาพในการดูแลตนเองในโรคเบาหวานของท่านโดยทีมดูแลสุขภาพ ซึ่งหน่วยบริการของท่านจะถูกสุ่มให้อยู่ 1 ใน 3 กลุ่ม อันได้แก่ กลุ่มทดลอง1 กลุ่มทดลอง2 และกลุ่มควบคุม โดยทั้ง 3 กลุ่มจะได้รับข้อมูลและการดูแลพื้นฐานเช่นเดียวกัน แต่แตกต่างกันในรูปแบบของการให้การอบรมในการดูแลตนเองของผู้ป่วยเบาหวาน ซึ่งจะใช้เวลานาน ไม่เกิน ครึ่งวัน โดย

For peer review only - http://bmjopen.bmj.com/site/about/guidelines.xhtml

- กลุ่มที่1 อบรมโดยบุคลากรทางการแพทย์ พยาบาล หรือ เจ้าหน้าที่สาธารณสุข
- กลุ่มที่2 อบรมโดยกลุ่มเพื่อนช่วยเพื่อนหรืออสม.ที่ผ่านการฝึกอบรมด้านการเพิ่มศักยภาพในการดูแลตนเองในโรคเบาหวาน
- กลุ่มที่ 3 ได้รับความรู้จากการดูแลตามมาตรฐานของกระทรวงสาธารณสุข

ซึ่งทั้งสามกลุ่ม จะมีการนัด หรือ ติดตาม ความก้าวหน้าพฤติกรรมการดูแลตนเอง ทุกๆ 1-3 เดือน

การติดตามผลเลือดและตอบแบบสอบถาม จะถูกทำในครั้งแรกข้างต้น และอีก 2 ครั้ง คือ ที่ 6 เดือนและที่ 12 เดือนนับจากครั้งแรกที่อบรม โดยทั้ง 3 ครั้ง ท่านจะถูกเจาะเลือดเพื่อส่งตรวจปริมาตรทั้งสิ้นครั้งละ 40 มิลลิลิตร

ความเสี่ยงจากการเข้าร่วมการวิจัยนี้

ความเสี่ยงจากการเจาะเลือด - ท่านอาจรู้สึกหน้ามืด เป็นลม ปวดบริเวณที่เจาะ หรือมีจ้ำเลือดบริเวณที่เจาะ มีความเสี่ยงน้อยมากที่จะเกิดการติดเชื้อจากการเจาะเลือดเพราะเราใช้เข็มเจาะเลือดที่ปราศจากเชื้อและใช้ครั้งเดียวทิ้ง การเจาะเลือดทั้งหมดอยู่ในสถานพยาบาล และหากมีปัญหาจะได้รับการดูแลทันที

ท่านจะได้ประโยชน์อะไรจากการศึกษานี้

ท่านจะได้รับความรู้เพื่อนำไปใช้ในการดูแลตนเองเพื่อควบคุมโรคเบาหวานของท่าน และผลสรุปที่ได้จากการศึกษานี้จะเป็นประโยชน์ต่อผู้ป่วยรายอื่นในอนาคต

ค่าใช้จ่ายในการเข้าร่วมวิจัย

ท่านจะไม่ต้องเสียค่าใช้จ่ายเพิ่มเติมเกี่ยวกับการวิจัยนอกเหนือจากค่ารักษาปกติที่ควรจะเป็นซึ่งท่านสามารถใช้สิทธิในการเบิกจ่ายได้ตามสิทธิการรักษาปกติของท่าน

ค่าตอบแทน

สำหรับการมาตรวจติดตามที่เพิ่มจากการตรวจรักษาตามปกติ จำนวน 5 ครั้ง ซึ่งจะมีการตรวจเลือด รวม 3 ครั้ง ท่านจะได้รับค่าเดินทางและค่าเสียเวลาครั้งละ 100 บาท (รวม 500 บาทต่อคน ตลอดระยะเวลาโครงการ 1 ปี)

For peer review only - http://bmjopen.bmj.com/site/about/guidelines.xhtml

หากท่านได้รับบาดเจ็บจากการเข้าร่วมการศึกษาวิจัย

หากท่านได้ปฏิบัติตามคำแนะนำของแพทย์ผู้วิจัยแล้ว กระบวนการต่าง ๆ ในการวิจัยทำให้ท่านได้รับบาดเจ็บ เมื่อผู้วิจัยได้รับแจ้งจากท่าน ท่านจะได้รับการส่งต่อเพื่อรักษาภาวะดังกล่าวทันที

ท่านจะทำอย่างไรหากท่านไม่ต้องการเข้าร่วมการศึกษาวิจัย หรือเปลี่ยนใจระหว่างร่วมศึกษาวิจัย

ท่านไม่จำเป็นต้องเข้าร่วมการศึกษาวิจัยนี้หากท่านไม่สมัครใจ หลังจากท่านตัดสินใจจะเข้าร่วมการศึกษาแล้ว ท่านสามารถจะถอนตัวได้ตลอดเวลา การตัดสินใจของท่านจะไม่มีผลต่อการรักษาในอนาคต หรือการดูแลอื่นใดหากท่านไม่ต้องการเข้าร่วมการศึกษาหรือต้องการหยุดการศึกษา ณ เวลาใดก็ตาม แพทย์ของท่านจะอธิบายให้ทราบถึงการรักษาอื่น ๆ ซึ่งเป็นทางเลือกที่มีอยู่ขณะนี้ ผู้วิจัยอาจจะตัดสินใจยกเลิกท่านจากการศึกษา หากเห็นว่าจะเป็นประโยชน์สำหรับท่านมากกว่า

ใครจะรู้บ้างว่าท่านเข้าร่วมการศึกษานี้

แพทย์ประจำตัวท่าน (แพทย์เวชปฏิบัติทั่วไป) ควรจะได้รับทราบว่าท่านตัดสินใจเข้าร่วมการศึกษาวิจัยนี้ ข้อมูลของท่านที่ถูกบันทึกไว้ระหว่างการศึกษา เช่นเดียวกับข้อมูลที่เกี่ยวข้องจากแฟ้มเวชระเบียนของโรงพยาบาล คลินิก บริษัทฯ หรือข้อมูลอื่น ๆ จะถูกเก็บไว้เป็นความลับตลอดเวลา คณะกรรมการจริยธรรมการวิจัยสามารถที่จะขอตรวจสอบข้อมูลเหล่านี้ได้ โดยข้อมูลเหล่านี้จะยังเก็บรักษาไว้เป็นเรื่องลับเฉพาะ

การปกป้องรักษาข้อมูล : ข้อมูลใดบ้างที่จะถูกเก็บรวบรวมไว้จากการศึกษานี้

ข้อมูลที่ถูกเก็บรวบรวมนั้นจะมีเฉพาะในส่วนที่เกี่ยวข้องกับการศึกษาเพื่อวัตถุประสงค์ทางการวิจัยทางการแพทย์ โดยจะไม่มีการอ้างถึงข้อมูลส่วนตัวของท่าน อันได้แก่ชื่อท่าน ในรายงานหรือวารสารใด ๆ หากท่านตกลงใจเข้าร่วมการศึกษา ท่านยินยอมที่จะไม่จำกัดการให้ข้อมูลที่เป็นส่วนตัวยกเว้นในกรณีที่ขัดต่อสิทธิส่วนบุคคลภายใต้กฎหมายคุ้มครองลิขสิทธิ์ส่วนบุคคล

หากท่านมีคำถามเกี่ยวกับการศึกษานี้ท่านสามารถติดต่อใครได้บ้าง

หากท่านมีคำถามหรือมีความวิตกกังวลเกี่ยวกับการศึกษาวิจัยนี้ หรือสงสัยว่าท่านกำลังได้รับบาดเจ็บจากการเข้าร่วมการวิจัยนี้ กรุณาติดต่อ คุณกุลญาภา อยู่นัด ได้ที่ ภาควิชาเวชศาสตร์ครอบครัว โทรศัพท์ที่ทำงาน 053-936362 (ในเวลาราชการ)

ส่วนแสดงความยินยอม

โดยการลงนามในหนังสือยินยอมฉบับนี้ ท่านยอมรับว่าได้อ่านเอกสารฉบับนี้แล้วและได้รับคำอธิบายเกี่ยวกับการศึกษาวิจัยนี้ รวมถึงได้รับคำตอบเกี่ยวกับข้อสงสัยต่าง ๆ ที่ท่านมีจากผู้วิจัยแล้ว

For peer review only

For peer review only - http://bmjopen.bmj.com/site/about/guidelines.xhtml

ท่านตกลงใจที่จะเข้าร่วมในการศึกษาวิจัยนี้ และมีสิทธิ์ที่จะถอนตัวจากการศึกษาวิจัยนี้ได้ทุกเมื่อ โดยไม่มีผลต่อการรักษาในอนาคต โดยการลงนามนี้ท่านไม่ได้สละสิทธิ์ใด ๆ ที่พึงมีทางกฎหมาย

ทั้งนี้ ท่านยินยอมที่จะเข้ารับการอบรมในโครงการวิจัย ตอบแบบสอบถามและ

ลายมือชื่ออาสาสมัคร ________________________________ วัน-เดือน-ปี________________

(__________________________________)

ลายมือชื่อเจ้าหน้าที่ผู้ให้ข้อมูล ________________________________ วัน-เดือน-ปี________________

ณ สถานพยาบาล

(__________________________________)

พยาน ________________________________ วัน-เดือน-ปี________________

(__________________________________)

Consent form WP3 intervention phase DSME 5 June 2018

For peer review only

SPIRIT

STANDARD PROTOCOL ITEMS: RECOMMENDATIONS FOR INTERVENTIONAL TRIALS

SPIRIT 2013 Checklist: Recommended items to address in a clinical trial protocol and related documents*

Section/item	ItemNo	Description	Page/Line number
**Administrative information**			
Title	1	Descriptive title identifying the study design, population, interventions, and, if applicable, trial acronym	1
Trial registration	2a	Trial identifier and registry name. If not yet registered, name of intended registry	Line 357
	2b	All items from the World Health Organization Trial Registration Data Set	-
Protocol version	3	Date and version identifier	Lines 357-358
Funding	4	Sources and types of financial, material, and other support	Lines 344-346
Roles and responsibilities	5a	Names, affiliations, and roles of protocol contributors	Page 1 and Lines 338-342
	5b	Name and contact information for the trial sponsor	Lines 359-360
	5c	Role of study sponsor and funders, if any, in study design; collection, management, analysis, and interpretation of data; writing of the report; and the decision to submit the report for publication, including whether they will have ultimate authority over any of these activities	Lines 344-353

For peer review only

	5d	Composition, roles, and responsibilities of the coordinating centre, steering committee, endpoint adjudication committee, data management team, and other individuals or groups overseeing the trial, if applicable (see Item 21a for data monitoring committee)	Lines 322-326
**Introduction**			
Background and rationale	6a	Description of research question and justification for undertaking the trial, including summary of relevant studies (published and unpublished) examining benefits and harms for each intervention	Page 4 and 5
	6b	Explanation for choice of comparators	Lines 99-103
Objectives	7	Specific objectives or hypotheses	Lines 92-94, 217-226
Trial design	8	Description of trial design including type of trial (eg, parallel group, crossover, factorial, single group), allocation ratio, and framework (eg, superiority, equivalence, noninferiority, exploratory)	Lines 98-103
**Methods: Participants, interventions, and outcomes**			
Study setting	9	Description of study settings (eg, community clinic, academic hospital) and list of countries where data will be collected. Reference to where list of study sites can be obtained	Lines 105-117
Eligibility criteria	10	Inclusion and exclusion criteria for participants. If applicable, eligibility criteria for study centres and individuals who will perform the interventions (eg, surgeons, psychotherapists)	Lines 119-129

For peer review only - http://bmjopen.bmj.com/site/about/guidelines.xhtml

Interventions	11a	Interventions for each group with sufficient detail to allow replication, including how and when they will be administered	Lines 157-187
	11b	Criteria for discontinuing or modifying allocated interventions for a given trial participant (eg, drug dose change in response to harms, participant request, or improving/worsening disease)	n/a
	11c	Strategies to improve adherence to intervention protocols, and any procedures for monitoring adherence (eg, drug tablet return, laboratory tests)	Lines 192-197
	11d	Relevant concomitant care and interventions that are permitted or prohibited during the trial	n/a
Outcomes	12	Primary, secondary, and other outcomes, including the specific measurement variable (eg, systolic blood pressure), analysis metric (eg, change from baseline, final value, time to event), method of aggregation (eg, median, proportion), and time point for each outcome. Explanation of the clinical relevance of chosen efficacy and harm outcomes is strongly recommended	Lines 217-229
Participant timeline	13	Time schedule of enrolment, interventions (including any run-ins and washouts), assessments, and visits for participants. A schematic diagram is highly recommended (see Figure)	Page 9-11

For peer review only - http://bmjopen.bmj.com/site/about/guidelines.xhtml

Sample size	14	Estimated number of participants needed to achieve study objectives and how it was determined, including clinical and statistical assumptions supporting any sample size calculations	Lines 140-148
Recruitment	15	Strategies for achieving adequate participant enrolment to reach target sample size	n/a
**Methods: Assignment of interventions (for controlled trials)**			
Allocation:			
Sequence generation	16a	Method of generating the allocation sequence (eg, computer-generated random numbers), and list of any factors for stratification. To reduce predictability of a random sequence, details of any planned restriction (eg, blocking) should be provided in a separate document that is unavailable to those who enrol participants or assign interventions	Lines 131-138
Allocation concealment mechanism	16b	Mechanism of implementing the allocation sequence (eg, central telephone; sequentially numbered, opaque, sealed envelopes), describing any steps to conceal the sequence until interventions are assigned	n/a
Implementation	16c	Who will generate the allocation sequence, who will enrol participants, and who will assign participants to interventions	n/a
Blinding (masking)	17a	Who will be blinded after assignment to interventions (eg, trial participants, care providers, outcome assessors, data analysts), and how	n/a

	17b	If blinded, circumstances under which unblinding is permissible, and procedure for revealing a participant's allocated intervention during the trial	
**Methods: Data collection, management, and analysis**			
Data collection methods	18a	Plans for assessment and collection of outcome, baseline, and other trial data, including any related processes to promote data quality (eg, duplicate measurements, training of assessors) and a description of study instruments (eg, questionnaires, laboratory tests) along with their reliability and validity, if known. Reference to where data collection forms can be found, if not in the protocol	Lines 228 Table 2
	18b	Plans to promote participant retention and complete follow-up, including list of any outcome data to be collected for participants who discontinue or deviate from intervention protocols	n/a
Data management	19	Plans for data entry, coding, security, and storage, including any related processes to promote data quality (eg, double data entry; range checks for data values). Reference to where details of data management procedures can be found, if not in the protocol	Lines 264
Statistical methods	20a	Statistical methods for analysing primary and secondary outcomes. Reference to where other details of the statistical analysis plan can be found, if not in the protocol	Lines 277-285
	20b	Methods for any additional analyses (eg, subgroup and adjusted analyses)	Lines 277-285

For peer review only - http://bmjopen.bmj.com/site/about/guidelines.xhtml

	20c	Definition of analysis population relating to protocol non-adherence (eg, as randomised analysis), and any statistical methods to handle missing data (eg, multiple imputation)	n/a
**Methods: Monitoring**			
Data monitoring	21a	Composition of data monitoring committee (DMC); summary of its role and reporting structure; statement of whether it is independent from the sponsor and competing interests; and reference to where further details about its charter can be found, if not in the protocol. Alternatively, an explanation of why a DMC is not needed	Lines 322
	21b	Description of any interim analyses and stopping guidelines, including who will have access to these interim results and make the final decision to terminate the trial	n/a
Harms	22	Plans for collecting, assessing, reporting, and managing solicited and spontaneously reported adverse events and other unintended effects of trial interventions or trial conduct	n/a
Auditing	23	Frequency and procedures for auditing trial conduct, if any, and whether the process will be independent from investigators and the sponsor	n/a
**Ethics and dissemination**			
Research ethics approval	24	Plans for seeking research ethics committee/institutional review board (REC/IRB) approval	Lines 364

Protocol amendments	25	Plans for communicating important protocol modifications (eg, changes to eligibility criteria, outcomes, analyses) to relevant parties (eg, investigators, REC/IRBs, trial participants, trial registries, journals, regulators)	Lines 319-320
Consent or assent	26a	Who will obtain informed consent or assent from potential trial participants or authorised surrogates, and how (see Item 32)	Lines 150-155
	26b	Additional consent provisions for collection and use of participant data and biological specimens in ancillary studies, if applicable	n/a
Confidentiality	27	How personal information about potential and enrolled participants will be collected, shared, and maintained in order to protect confidentiality before, during, and after the trial	Lines 150-155
Declaration of interests	28	Financial and other competing interests for principal investigators for the overall trial and each study site	Lines 362-363
Access to data	29	Statement of who will have access to the final trial dataset, and disclosure of contractual agreements that limit such access for investigators	Lines 348-349
Ancillary and post-trial care	30	Provisions, if any, for ancillary and post-trial care, and for compensation to those who suffer harm from trial participation	n/a

For peer review only - http://bmjopen.bmj.com/site/about/guidelines.xhtml

Dissemination policy	31a	Plans for investigators and sponsor to communicate trial results to participants, healthcare professionals, the public, and other relevant groups (eg, via publication, reporting in results databases, or other data sharing arrangements), including any publication restrictions	Lines 328-336
	31b	Authorship eligibility guidelines and any intended use of professional writers	n/a
	31c	Plans, if any, for granting public access to the full protocol, participant-level dataset, and statistical code	n/a
**Appendices**			
Informed consent materials	32	Model consent form and other related documentation given to participants and authorised surrogates	Attached
Biological specimens	33	Plans for collection, laboratory evaluation, and storage of biological specimens for genetic or molecular analysis in the current trial and for future use in ancillary studies, if applicable	n/a

*It is strongly recommended that this checklist be read in conjunction with the SPIRIT 2013 Explanation & Elaboration for important clarification on the items. Amendments to the protocol should be tracked and dated. The SPIRIT checklist is copyrighted by the SPIRIT Group under the Creative Commons "Attribution-NonCommercial-NoDerivs 3.0 Unported" license.

For peer review only - http://bmjopen.bmj.com/site/about/guidelines.xhtml
